# Bacteriophages in the Control of *Aeromonas* sp. in Aquaculture Systems: An Integrative View

**DOI:** 10.3390/antibiotics11020163

**Published:** 2022-01-27

**Authors:** Carla Pereira, João Duarte, Pedro Costa, Márcia Braz, Adelaide Almeida

**Affiliations:** Department of Biology and CESAM, Campus Universitário de Santiago, University of Aveiro, 3810-193 Aveiro, Portugal; j.macedoduarte@ua.pt (J.D.); pedrommrscosta@ua.pt (P.C.); marciabraz97@ua.pt (M.B.)

**Keywords:** phage therapy, *Aeromonas* species, bacterial infections, aquaculture, fish

## Abstract

*Aeromonas* species often cause disease in farmed fish and are responsible for causing significant economic losses worldwide. Although vaccination is the ideal method to prevent infectious diseases, there are still very few vaccines commercially available in the aquaculture field. Currently, aquaculture production relies heavily on antibiotics, contributing to the global issue of the emergence of antimicrobial-resistant bacteria and resistance genes. Therefore, it is essential to develop effective alternatives to antibiotics to reduce their use in aquaculture systems. Bacteriophage (or phage) therapy is a promising approach to control pathogenic bacteria in farmed fish that requires a heavy understanding of certain factors such as the selection of phages, the multiplicity of infection that produces the best bacterial inactivation, bacterial resistance, safety, the host’s immune response, administration route, phage stability and influence. This review focuses on the need to advance phage therapy research in aquaculture, its efficiency as an antimicrobial strategy and the critical aspects to successfully apply this therapy to control *Aeromonas* infection in fish.

## 1. Introduction

Increasing global production to offset progressive worldwide reductions in the amount and quality of natural seafood populations has contributed to making aquaculture one of the fastest-growing productive agricultural sectors. In the last few years, aquaculture rapidly expanded its driving economic growth and contributed to global food security [1]. In 2018, almost 38% of all fish caught or farmed worldwide were traded on international markets, generating a total value of 164 billion USD [1]. However, aquaculture industries often suffer extensive financial losses due to uncontrolled microbial diseases, threatening their sustainability and growth [2]. The main biological agents responsible for waterborne diseases include bacteria, viruses, protists, helminths, fungi and oomycetes [2,3,4]. However, bacteria are the main cause of infections and the major concern in the aquaculture industry, leading to large financial losses that endanger the sector’s sustainability and causing heavy losses to fish farming plants [2,3,4,5]. The main bacterial diseases in aquaculture are vibrosis, aeromonasis, edwardsiellosis, pseudomonasis, flavobacteriosis, mycobacteriosis, streptococcosis, renibacteriosis, infection with anaerobic bacteria (*Clostridium botulinum* and *Enterobacterium catenabacterium*) and intracellular bacterial infection (*Francisella noatunensis*, *Piscirickettsia salmonis*, *Hepatobacter penaei*, *Chlamydia* spp.) [6,7,8]. Aeromonasis in fish is caused by *Aeromonas* species, namely *A. hydrophila*, *A. salmonicida*, *A. caviae*, *A. sobria*, *A. veronii* and *A. jandaei* [9,10,11]. These species are common in freshwater habitats and are frequently associated with severe infections and mortality in various freshwater and marine fish [12,13,14,15].

Disease prevention in aquaculture species includes several strategies and management solutions, including vaccines and antibiotics. Vaccination is considered crucial as it is one of the main approaches to prevent and control diseases in aquaculture [16]. Currently, there are over 26 vaccines approved for a variety of fish species [6,17,18,19,20]. This set of vaccines has proven to successfully protect fish against a variety of severe diseases [20]. Most vaccines are based on inactivated microorganisms and adjuvants delivered through immersion or injection [20]. Live vaccines are more efficient because they generate a strong antibody response by mimicking the natural pathogen infection and have a greater potential to be administered via oral or by immersion [20]. Prophylactic immunization for bacterial diseases in farmed fish has been attempted with some success against *Yersinia ruckeri* and *A. salmonicida* [21]. Despite some successes, there are no vaccines available for many of the pathogens [2,22,23,24]. Moreover, the vaccines that exist, do not always offer thorough protection nor can they be used to protect juvenile fish, which lack a mature immune system [24]. In addition, the development of new fish vaccines can be expensive and vaccination is impractical in small animals such as the fish larvae, which are usually more susceptible to disease, and cannot develop specific immunity [2,22,23,24].

To overcome these constraints, aquacultures have resorted to antimicrobial drugs to treat bacterial fish diseases or as a preventive measure administered in feeds [25]. This results in environmental leakage of these chemicals and selection of resistance. The prolonged presence of antibiotics, even at concentrations lower than the minimum inhibitory concentration, in the polybacterial matrices of ponds, sediments, or biofilms can provoke a selective pressure on the bacterial populations and the consequential exchange of antimicrobial genes between bacteria [25,26,27,28]. The transition of antimicrobial residues, antimicrobial-resistant bacteria and resistance genes from the aquatic environment to terrestrial livestock and humans presents an increased risk of wide-spreading drug resistance [29]. The emergence of antibiotic-resistant *Aeromonas* strains in aquaculture has been observed [30,31], making it urgent to find alternative control methods to treat these infections. Phage therapy appears as an efficient, environmentally friendly and empirical solution to control pathogenic bacteria in farmed fish.

Phages are bacteria-infecting viruses that are abundantly present in the environment and essential in controlling bacterial populations in natural systems [2,32]. Their potential use in agriculture, aquaculture, veterinary, food safety and medicine is being studied worldwide [2,33,34,35,36,37,38,39,40,41,42,43,44,45,46,47,48,49]. In recent years, the use of phages to inactivate pathogenic bacteria in farmed fish has gained momentum, mainly due to their inherent low toxicity [32]. This growing trend seems to show an increased interest in industrial applications of phages in aquaculture. However, only one phage-based product, called BAFADOR^®^, was approved for use to control *Pseudomonas* and *Aeromonas* in aquaculture [50].

This review highlights and discusses the potential that phage therapy has to control *Aeromonas* in fish, the main preventive measures used and the aspects that need to be considered when applying phage therapy in aquaculture.

## 2. *Aeromonas* Infections

The fish farming industry is heavily affected by pathogenic bacteria infections that can become one of the main sources of financial loss [2,51,52]. Unfavorable conditions found in fish farms, such as high fish density, high temperatures, rapid growth, overfeeding and residue accumulation, increase the possibility of pathogen transmission between individuals and can consequently provoke disease outbreaks. Furthermore, when sick or dead fish are not extracted from the farming area in time, the risk of disease increases, allowing pathogens to become more aggressive in the already polluted environments. It has been shown that pathogenic microorganisms are introduced by wild fish into the aquatic environment and not farmed fish, as was previously thought [2].

The genus *Aeromonas* (phylum, Proteobacteria; class, γ-Proteobacteria; order, Aeromonadales; and family, *Aeromonadaceae*) comprises a group of Gram-negative bacteria, widely distributed in aquatic environments, being easily isolated from animals such as fish and crustaceans [53,54,55], and comprises a total of 36 species that are currently described in the genus [53]. The members of the genus *Aeromonas* can be split into two groups based on their biochemical characteristics and growth conditions: psychrophilic, composed of non-motile bacteria with optimal growth between 22–25 °C; and mesophilic, composed of mobile bacteria with a single polar flagellum in most of the species and an optimal growth at 35–37 °C [53,54,55]. Table 1 summarizes the general characteristics of the genus *Aeromonas* members. The ability to adapt enables *Aeromonas* to colonize terrestrial environments and their inhabitants, allowing them to be found in sources, such as soils, plants, fruits, vegetables, birds, reptiles, amphibians, among others [54]. Some species can cause disease in humans, fish and other aquatic animals. Infectious processes usually develop in immunocompromised hosts; however, in fish and other marine animals, virulent strains have already been reported [11,56,57]. Although the way how these pathogens are transmitted is still unclear, species such as *Aeromonas* (e.g., *A. hydrophila*, *A. caviae*, and *Aeromonas dhakensis*) are well known as causative agents of human diseases, including gastroenteritis, soft tissue infections, septicemia, peritonitis, pneumonia and diarrhea [58,59]. *A. caviae*, *A. hydrophila, A. sobria*, *A. salmonicida*, *A. jandaei*, *Aeromonas bestiarum* and *A. veronii* are typically associated with fish diseases and mortality [9,10,11]. Supplementary Materials Table S1 lists the common *Aeromonas* sp. that are detected in fish. Motile *Aeromonas*, such as pathogenic bacteria, can be responsible for fish deaths up to 80–100% within 1–2 weeks, leading to substantial economic losses in commercial carp farming due to high mortality rates and producing quality degradation [60,61,62]. The main species affected by disease and death caused by these pathogens are carp, tilapia, rainbow trout, brown trout, eel, perch, catfish, goldfish and salmon [63]. *Aeromonas* virulence is complex because several factors significantly contribute to the development of the infectious process as the effectiveness of the host immune system decreases [5,64]. Structural components, toxins and extracellular products, acting jointly or individually, enable these microorganisms to colonize and infect hosts [9,54,65,66]. Virulence factors can be expressed differentially between species, making some strains more virulent than others [54]. Some of the most relevant fish diseases that result in major die-offs and fish kills and are caused by the above factors are: external ulcerative lesions, fin and tail rot, red sores, ocular ulceration, anal region pale body colour, reddish head, fin haemorrhagic, septicaemia, hemodiapedesis, anorexia, exophthalmos and erythrodermatitis, revealed clear ascites, haemorrhages and destruction of sheathed tissues in spleen and renal tubular necrosis in the kidney, liver congestion, enlargement of spleen and kidney and enteritis [67,68,69]. *Aeromonas* infection signs and symptoms may vary depending on the location of the infection and the type of bacteria (Table S1).

*A. salmonicida* and *A. hydrophila*, the most studied species in aquaculture within the *Aeromonas* genus, are relevant fish pathogens in aquaculture, responsible for significant economic losses worldwide due to high mortality and morbidity in several fish species.

*A. salmonicida* is one of the main pathogens responsible for furunculosis in wild and cultured salmonids, causing bacterial septicemia in fish [55,70], and can infect several fish species such as Arctic charr (*Salvelinus alpinus*), Atlantic cod (*Gadus morhua*), Atlantic halibut (*Hippoglossus hippoglossus*), Atlantic salmon (*Salmo salar*), Atlantic wolffish (*Anarhichas lupus),* carp (*Cyprinus carpio*), Chinese perch (*Siniperca chuatsi*), flounder (*Platichthys flesus*), goldfish (*Carassius auratus*), lumpsucker (*Cyclopterus lumpus*), rainbow trout (*Oncorhynchus mykiss*), spotted wolfish (*Anarhichas minor*) and turbot (*Scophthalmus maximus*) [70,71,72,73]. Although *A. salmonicida* is only regarded as a primary pathogen in fish and not in humans because of its inability to grow at 37 °C, several studies have demonstrated its ability to cause human infections that result in septicemia and endocarditis [74,75,76].

**Table 1:** List of some general characteristics of members of the genera *Aeromonas*.

Characteristic	Description	References
Habitat	Distributed in aquatic environments, usually isolated freshly from different water sources (sea, reservoirs and sewage). Some species can be isolated from healthy and diseased fish, chironomid egg masses and intestinal/extraintestinal human samples.	[53,77]
General morphological characteristics	Gram-negative bacilli	[53,77]
General biochemical characteristics	Some species have mobility (e.g., *A. hydrophila*, *A. caviae* and *A. veronii*) Facultative anaerobes; Oxidase positive; Catalase positive; Capable of degrading nitrates to nitrites, glucose fermenters; Resistant to vibriostatic agent O/129 (2,4-diamino-6,7 diisopropylpteridine) at concentrations of 150 mg/disc with few exceptions (*Aeromonas australiensis* and *Aeromonas cavernicola* and a few *Aeromonas eucrenophila* and *A. veronii* strains).	[53,77]
Isolation and cultivation media	General: Tryptic soy agar (TSA) and Tryptic Soy Broth (TSB). Specific: Starch-ampicillin agar; Taurocholate-tellurite-gelatin agar; Ampicillin dextrin agar; Cefsulodin-irgasan-novobiocin agar, MacConkey agar and blood agar enriched with ampicillin; Glutamate starch phenol red and Aerosmart AH medium.	[54,58,78,79,80,81,82]
NaCl tolerance	*Aeromonas* can tolerate up to 5% NaCl for growth.	[83,84]
Optimum growth temperature	*Aeromonas* grow best at temperatures between 22 °C and 37 °C, depending on the strain under analysis. Psychrophilic *Aeromonas* (e.g., *A. salmonicida*), grow at temperatures lower than 22–25 °C. Mesophilic *Aeromonas* (e.g., *A. caviae*, *A. hydrophila*, *A. veronii*), grow at temperatures between 35–37 °C. Survive in low temperatures (2–10 °C).	[83]
Optimum growth pH	Survive at pH = 5	[84]
Virulence factors and pathogenicity	Structural components (e.g., flagella, pili, proteins and membrane antigens). Extracellular products: (e.g., hemolysin, protease, lipase, protease, DNases cytotoxic enterotoxin) Secretion systems: Type II secretion system Type III secretion system Type IV secretion system Type VI secretion system	[54,85,86]

*A. hydrophila* is an opportunistic pathogen with a wide host range (e.g., amphibians, birds, fish, reptiles and mammals) [87,88] and is responsible for several bacterial diseases that have caused the loss of millions of dollars in the global freshwater aquaculture industry [6,63,89]. *A. hydrophila* can infect several freshwater and marine fish, causing “Motile *Aeromonas* Septicemia (MAS)”, or “Red-Sore Disease” [90], which results in lesions, scale shedding, gill and anal hemorrhage, abdominal swelling, skin ulcers and septicemia [91]. Different fish species, including tilapia (*Oreochromis niloticus*), catfish (*Ictalurus punctatus*), striped catfish (*Pangasianodon hypophthalmus*), goldfish (*C. auratus),* common carp (*C. carpio*) and eel (*Anguilla* spp.) are affected by *A. hydrophila*. The high mortality, weight loss and high treatment costs lead to severe economic losses to the aquaculture industry [6,92,93,94,95,96].

## 3. Disease Control and Alternative Approaches

Sustainably preventing aquaculture diseases is desirable, but not always possible since supplying the optimal conditions and feeding can be economically challenging. Furthermore, its success may require effective biocontrol techniques to reduce infections [97,98]. The rapid spread and the ubiquitous nature of fish pathogenic microorganisms mean that infection control and prevention can be difficult [99,100]. Preventing and controlling diseases in aquaculture becomes more challenging with: (1) severe fecal contamination in fish farm waters [101,102], because few medications are licensed for use in fisheries [2,25] and many chemotherapeutic agents are ineffective against endospores and zoospores, leading to treatment failure in the case of infection [103,104]; (2) irregular environmental conditions (e.g., elevated temperatures, salinity variations, decreased oxygen concentrations, high organic load) that may contribute to disease outbreaks, often weakened by the sensitive fish’s innate defense system [98,102,105]; (3) high fish densities (greater than the indicated for each species), common practice in farming systems, which reduces infection resistance [106]; (4) different stages of the fish life cycle, that affect the development of the immune system, increases the frequency of infections [106,107]; (5) the indiscriminate and prophylactic use of antibiotics that increases the resistance problem in common pathogenic bacteria and the concern with the antibiotic spread in the environment [25,28,108].

Despite the growing concern about the emergence of antimicrobial resistance in bacteria, pathogen control in aquaculture is still mostly reliant on antibiotic usage [55]. It is difficult to know exactly how much antibiotics are used in aquaculture [109] since it depends on the antibiotics, the authorized limits in each country, the various farming types and the diversity of aquaculture species [110,111,112,113]. A drastic reduction in antibiotic use has been seen in recent years in some countries due to vaccination and improved husbandry practices [112,113,114,115], particularly in Norway [116]. However, antibiotics were and are abusively applied in some countries, such as China, Vietnam and India, to promote growth, as well as to treat and prevent diseases [39,117,118]. According to Lulijwa et al. (2020), 73% of the main aquaculture producing countries use florfenicol, sulphadiazine and oxytetracycline and 55% applied amoxicillin, erythromycin, enrofloxacin and sulfadimethoxine [108]. The United States (USA), the European Union (EU) and Japan, have strict regulations on the use of antibiotics and restrict minimum limits approved in aquaculture. [25]. In 2006, the EU banned the use of antibiotics as growth promoters in farm animals [119]. The U.S. Food and Drug Administration (FDA) only allows the use of florfenicol, oxytetracycline and sulfadimethoxine/ormetoprim in the aquaculture industry [25,113]. The United Kingdom (UK) only allows amoxicillin, cotrimazine, oxolinic acid, oxytetracycline and sarafloxacin [25,113]. China and Vietnam, on the other hand, are the main consumers of antibiotics, which might explain their rampant prophylactic use [108]. The Chinese government currently allows the use of 13 antibiotics (doxycycline, enrofloxacin, florfenicol, flumequine, neomycin, norfloxacin, oxolinic acid, sulfamethazine, sulfamonomethoxine, thiamphenicol and trimethoprim) [108,110]. Until 2014, 30 antibiotics were authorized in Vietnam. But even though they also banned ciprofloxacin and fluoroquinolones in 2016, their presence is still detected in later studies. While Vietnam and China have relatively big domestic markets, the other Asian countries, such as India, Thailand, South Korea and Bangladesh, have to use fewer antibiotics to meet the strict regulations of their trading partners, namely the USA, the EU and Japan [108,120,121]. The antibiotics used in aquaculture production by the 15 major producers (2008 to 2018) have been described by Lulijwa et al. (2020) [108].

The regular and massive use of antibiotic prophylaxis in aquaculture systems has resulted in the emergence of multidrug-resistant bacteria, including *Aeromonas* resistant strains, making any antibiotic treatment ineffective in several fish such as catfish, koi carp and tilapia [122]. Furthermore, most antibiotic resistance studies have been conducted on these pathogens because of their unusual biofilm formation and antibiotic resistance [123]. The first fish pathogen reported that showed antimicrobial resistance was *A. salmonicida* and was resistant to tetracycline and sulfathiazole [124]. Jacobs and Chenia (2007) observed high levels of resistance to tetracycline (78.3%), amoxicillin (89.2%) and augmentin (86.5%) in *Aeromonas* isolates from trout, tilapia and koi from South African aquaculture systems [125]. *A. hydrophila* and *A. salmonicida* isolates exhibited higher resistance levels to different antimicrobial agents when compared with *Aeromonas encheleia*, *Aeromonas popoffii*, *A. veronii*, *Aeromonas media* and *Aeromonas ichtiosoma* isolates [125]. This is likely because large amounts of antibiotics, such as oxytetracycline, quinolones and trimethoprim, have been used over the years to treat furunculosis in infected salmonids [126]. In the last two decades, there has been an increase in reports of quinolone resistance among fish-associated aeromonads [10,127,128,129].

Vaccination is an alternative and feasible control method to prevent *Aeromonas* sp. infections; however, the vaccines available in aquaculture are still very limited. Vaccines have been successfully used in aquaculture, reducing the use of antibiotics, particularly in salmon production [39]. In aquacultures, the most currently used vaccines are inactivated vaccines since this present greater biosafety and are easier to license [130]. Live or attenuated vaccines have shown great potential, achieving fish immunization with a single dose and having low production costs [20,131]. However, the use of live bacteria poses a threat to the environment and therefore, few of these are licensed for commercial use [130,131]. Currently, over 26 fish vaccines are licensed and commercially available for use in various fish species [6,17,18,19,20] and have successfully protected fish against several fish diseases [20]. Most licensed vaccines contain inactivated microorganisms and adjuvants that can be delivered through immersion or by injection [20,130]. Unfortunately, vaccines for many farmed fish species and pathogenic bacteria have not been developed [39]. Duff (1942) was the first to report the application of vaccines against *A. salmonicida* in cutthroat trout, *Oncorhynchus clarki*. In these trials, the fish were fed inactivated *A. salmonicida* [132]. The development of the first salmonid vaccines that were delivered by immersion used the same bacterial inactivation principle applied in the Atlantic salmon (*S. salar*) [133]. However, these injection-based bacterial vaccines were not effective against *A. salmonicida* in Atlantic salmon as Bricknell and colleagues reported [134]. In this study, the extracellular polysaccharide vaccines induced an antibody response and were protective for about 2 months following injection [134]. In the last few years, several vaccines against typical *A. salmonicida* strains were developed to provide long-lasting protection in commercial salmonid culture [55,135]. In 2011, China granted the national class I new veterinary drug certificate to a killed whole-cell vaccine for *A. hydrophila* (J-1 strain), the first aquatic bacterial vaccine for this species [136]. AQUAVAC^®^ FNM is a non-mineral, oil-based injectable vaccine that contains two strains of *A. salmonicida*, the causative agent of furunculosis in Atlantic salmon [137]. Alpha Ject Panga 2 was approved in 2017 in Vietnam. The Alpha Ject Panga 2 is an injectable vaccine that protects against *A. hydrophila*, and *Edwardsiella ictaluri* [138]. DNA vaccines using carbon nanotubes or those that are yeast-based have, similar to inactivated vaccines, recombinant protein vaccines and bacterial lysates, demonstrated to stimulate protection against *A. hydrophila* [131,139,140,141,142]. The development of commercial vaccines against *A. hydrophila* in fish has been challenging because of its biochemical and serological heterogeneity [143]. Despite vaccination representing an effective strategy to prevent *Aeromonas* infections, these have been linked to a variety of side effects such as impaired growth, inflammation, fibrous adhesions in internal organs, scarification and pigment deposition [144,145,146,147]. Moreover, vaccines require developed and functional immune systems which, in larval or fry stages, will have low to poor outcomes [23]. Furthermore, they do not always offer full protection and can be very difficult to administer by injection [6]. As such, the application of virulent phages to prevent and/or treat infections appears as a promising strategy [51,91,94,148,149,150], at a time when more efficient approaches are needed to control *Aeromonas* diseases.

## 4. Therapeutic Application of Phages

Phage therapy uses phages, viruses that only infect prokaryotes (bacteria and archaea) [151,152], to inactivate pathogenic bacteria. They do not possess host-independent metabolism and cannot produce proteins, as such, are incapable of self-replication [153,154,155]. Phages were discovered independently by Frederick W. Twort in England in 1915 and by Felix d′Herelle in Paris in 1917 [156]. However, their phage application ideas were later abandoned by Western European countries due to the success of antibiotics [32,157]. It was only in recent decades that its interest was regained following the growing concern with antimicrobial resistance [32,42]. The emergence of pathogenic bacteria resistant to antibiotics has recently motivated the Western scientific community to re-evaluate phage therapy for the treatment of bacterial infections. To do so, several aspects of phage ecology, namely, abundance, viral decay rates, repair mechanisms, lysogeny and impact on bacterial communities, need to be further understood [158,159]. Since the regulatory acceptance of ListShield™ [produced by Intralytix Inc (Baltimore, MD, USA)], the first phage-based product (a cocktail of six different virulent phages approved to control *Listeria* in meat and poultry products), the amount of research and development of new phage-based technologies for pathogen control has increased [37,160]. Currently, the potential use of phage therapy in medicine [33,44,154,161,162], aquaculture [2,38,39,40,41,51,52], food safety [32,34,36,37,163,164,165,166], agriculture [34,35,167,168,169,170], veterinary [45,46,48] and wastewater treatments [171,172] has started to be studied worldwide.

As soon as they were discovered by Twort and d’Herelle in the early 1920s, phages were described as antibacterial agents for both humans and animals [173]. More than fifty years later, studies were also initiated using phages to control pathogenic bacteria in aquaculture. Following the first reported application of phages to control *A. hydrophila* in aquaculture, in 1981 [174], several virulent phages that infect the main bacterial pathogens present in aquacultures, such as *Aeromonas* spp., *Edwardsiella* spp., *Flavobacterium* spp., *Pseudomonas* spp. and *Vibrio* spp. [92,175,176,177,178,179,180,181,182,183,184,185,186,187,188,189] were isolated and characterized for potential therapeutic. Recent studies focused on the isolation and characterization of lytic phages, cocktails [95,150,184,185,190,191,192,193] and addressed the potential application of phages as a therapeutic agent to control diseases in aquaculture, their dosage and administration [194].

The prophylactic application of phages has been effective in treating *Edwardsiella tarda*, a bacterium that causes edwardsiellosis in loach *Misgurnus anguillicaudatus* [195]; *Lactococcus garvieae* causing lactococcosis in yellowtail *Seriola quinqueradiata* [196]; *Pseudomonas plecoglossicida* causing pseudomonosis in *Plecoglossus altivelis* [197,198], *Flavobacterium psychrophilum* and *Flavobacterium columnare* causing flavobacteriosis in *O. mykiss* [3,194,199,200]; as well as *Vibrio parahaemolyticus* and *Vibrio splendidus* infections in sea cucumber *Apostichopus japonicus* [192,201]. Other successful studies have previously reviewed phage therapy in aquaculture [2,39,40,202].

Over the last couple of years, few companies invested in phage-based solutions to control and/or prevent bacterial infections in aquaculture, as seen in the small number of products developed so far (Table 2).

## 5. Application of Phages Infecting *Aeromonas* sp. in Aquaculture

*Aeromonas* species are recognized as the third most targeted aquatic bacterial pathogen in phage application research [39] and were the first target for phage therapy in aquaculture back in 1981 [174]. These authors isolated eight *A. hydrophila* phages from which AH1 was selected for the study of biological control of disease in loach *M. anguillicaudatus*. The authors injected the loach *M. anguillicaudatus* with *A. hydrophila* and observed that, after 3 h of phage treatment, the bacterium had completely lost its infectivity and mortality halted in the phage inoculated animals. Even at a multiplicity of infection (MOI) of 0.001, infectivity and mortality were reduced to 40% of uninfected *A. hydrophila* [174]. Since then, several studies have evaluated and shown the promising results that some *Aeromonas* phages have as alternative biocontrol agents and their therapeutic (or prophylactic) potential in aquaculture. However, studies with phages aiming to prevent or eliminate *Aeromonas* spp. in aquaculture have been restricted to two pathogenic bacterial species so far, *A. hydrophila* and *A. salmonicida (*Tables S2 and S3).

Several studies have analyzed phenotypic and genotypic characterization, and evaluated the effectiveness of phages against *A. hydrophila*, including phages pAh1-C and pAh6-C [209]; Ahp1 [210]; pAh-1 [211]; Φ2 [95]; AP1, AP2, AP3 and AP4 [92]; CT45P and TG25P [212]; MJG [91], AHP-1 [181]; Akh-2 [149], PVN-02 [148,213], AhyVDH1 [214] and pAh6.2T [94] (Tables S2 and S3). Previous studies have shown that phages can be used to biocontrol *A. hydrophila* infections in loach (*M. anguillicaudatus*) [149,174,209], Nile tilapia (*O. niloticus*) [92,93], striped catfish (*P. hypophthalmus*) [95,148] and rainbow trout (*O. mykiss*) [91,94] (Table S3).

The first application of phages to control *A. hydrophila* in loach occurred in 1981 [174]. More than three decades later, Jun et al. (2013) showed that a single administration of simple suspensions of phages pAh1-C or pAh6-C increased survival rates against *A. hydrophila* infection. However, phage pAh6-C controlled *A. hydrophila* infection more effectively than phage pAh1-C [209]. Recently, Akmal and colleagues showed the protective effects, with increased survival rates (0–43%) and mean times to death in *M. anguillicaudatus* infected with *A. hydrophila* [10^7^ colony-forming units (CFU)/mL)] and treated with phage Akh-2 [10^8^ plaque-forming units (PFU)/mL)] [149]. The protective effect of *Aeromonas* phages to control *A. hydrophila* in Nile tilapia (*O. niloticus*) was also reported by [92,93]. El-Araby et al. (2016) applied a mixture of two phages by immersion to control *A. hydrophila* in Nile tilapia (*O. niloticus*) and reduced the mortality rate from 68% to 18% after a 15-day treatment. In another study, Hassan et al. (2018) showed the promising effect of phage AP2 to treat motile *Aeromonas* septicemia induced by *A. hydrophila* in Nile tilapia [92]. Only very recently, with Le et al. (2018), have phages been investigated for their potential to prevent and treat bacterial diseases in catfish (*P. hypophthalmus*). Namely, the ability that phages Φ2 and Φ5 have to inactivate and control *A. hydrophila* in striped catfish by injection and the observed cumulative mortality of fish decreases with the increase in MOI (cumulative mortality of 0%, 45% and 68% with an MOI of 100, 1 and 0.01) [95]. Dang and co-workers had similar results and observed that fish mortality depends on the phage dose used during treatment [148]. However, the difference is not so great, as that observed in Le et al. (2018) [95]. Dang et al. (2021) demonstrated that the relative survival percentage of catfish with *A. hydrophila* was 75.6–87.8% when fed with phage PVN02-sprayed feed [148]. Cao et al. (2020) administered phage MJG by injection, immersion and oral administrations to control *A. hydrophila* in rainbow trout and achieved a relative percent survival of 100%, 66.7%, and 50%, respectively [91]. Additionally, Dien et al. (2021) showed that treatments with phage pAh6.2TG significantly improved survivability of Nile tilapia exposed to lethal doses of *A. hydrophila* (10^7^ CFU/mL), with relative percent survival of 73.3% and 50% at an MOI of 1.0 and 0.1, respectively [94].

Several studies have shown the antimicrobial efficacy of different phages (including phage cocktails) to biocontrol *A. salmonicida* strains in vitro and/or in vivo experiments [51,150,180,215,216,217,218,219] (Tables S2 and S3). Among them, therapeutic (or prophylactic) applications of phages to control *A. salmonicida* in brook trout (*Salvelinus fontinalis*) [218], Atlantic salmon (*S. salar*) [219], rainbow trout (*O. mykiss*) [220] and Senegalese sole (*Solea senegalensis*) [51] (Table S3). Although Verner-Jeffreys et al. (2007) did not find any protective effects against *A. salmonicida* in Atlantic salmon treated with the mixture of phages O, R and B [219], three other studies showed clear differences between the phage-treated and control groups [51,218,220]. Imbeault et al. (2006) showed that phage HER110 delayed the onset of furunculosis by 7 days with fish mortality rates reducing from 100% to 10% after 45 days [218]. In 2015, Kim and colleagues showed notable positive effects, with increased survival rates (0–26%) and mean times to death in rainbow trout infected with *A. salmonicida* subsp. *salmonicida* (2.5 × 10^2^ CFU/fish) and treated with phage PAS-1 (2.4 × 10^6^ PFU/fish) [220]. The same was also verified in Senegalese sole (*S. senegalensis*) treated with phage AS-A, showing a significantly reduced mortality (36% to 0%) [51].

From the existing alternatives, phages have shown their potential to control *A. hydrophila* and *A. salmonicida*, but with some limitations. Their advantages and limitations in aquaculture have been thoroughly described in previous reviews [2,38,39,40,202,221]. Nevertheless, in this review, we highlight the major challenges phage application for *Aeromonas* sp. biocontrol face in the aquaculture industry and how they can be overcome.

## 6. Challenges Associated with the Use of Phages to Control *Aeromonas* sp.

Aquaculture studies have shown that phage therapy can grant fish overall protection and provided us with an optimistic outlook on the benefits that these phage-based technologies have to treat diseases in aquaculture. However, this therapy is still mostly in an early stage and needs to be further studied and described. Several studies have reported the isolation and characterization of new phages (Table S2) and their efficiency to control *A. hydrophila* and *A. salmonicida* (Tables S2 and S3); however, they are mainly in vitro with few reporting in vivo studies (Table S3). One of the issues facing the study of phage therapy is the ability to demonstrate its viability in vivo and in field conditions [51,52,198], because, in vitro assays are not enough to understand phage–bacteria interactions that occur in vivo.

While it is known that page therapy can prevent and treat infectious diseases, a few constraints may still hamper its application in aquaculture. Phage therapy requires a detailed understanding of bacteria–phage kinetics, time, phage and dosage, and application methods (e.g., oral administration through feed, injection or immersion). Some phage advantages (such as the narrow host range) can become drawbacks when designing phage therapy and should be well understood. Additionally, their ability to survive under environmental conditions is highly diversified, which makes it important to understand the complex problem of phage sensitivity to external abiotic factors (such as salinity, temperature, pH and UV radiation).

Several studies still overlook phage genome sequencing. The knowledge of how the natural mechanisms contribute to the emergence of phage-resistant bacteria and which bacterial receptors may be specific to a phage is crucial for the successful application of phages in aquaculture [222].

In addition, despite certification by the regulatory entities, the stigma producers and consumers have regarding phage safety must be addressed and overcome [207]. While the scientific interest regarding the industrial application of phages has been rising, few are the private companies that have followed the trend, started working on or have launched, phage-based technologies for aquaculture [39]. Therefore, additional efforts are needed to assess producer and consumer understanding, followed by educational campaigns to raise awareness and acceptance of phage application in aquaculture.

### 6.1. Phage Selection

The selection of appropriate phages is one of the main steps required to achieve successful phage-mediated control of pathogenic bacteria. The key pre-requisites during preparation and selection of phage suspensions to be used in phage therapy, include (i) lytic activity (only virulent phages should be used), (ii) host range, (iii) adsorption rate, (iv) growth parameters (burst size and latent period), (v) environmental stability, (vi) bacterial inactivation efficiency and (vii) safety [2,32,152,190,223].

Phages are non-toxic and there is little evidence of harmful phage immune responses. Nonetheless, it is still crucial that phage preparations are pure and free of bacterial components [224].

During phage isolation from environmental sources, only virulent phages should be selected for therapeutic application. Temperate phages should not be used in phage therapy [225] because they can grant the host immunity against the same or similar phages. The bacterial host can acquire new genetic traits like phage-encoded toxins and antibiotic resistance determinants by phage conversion since phages will easily convert the bacterial hosts into lysogens (phage-resistant), thus rendering them unable to cause immediate lysis [155,225]. Lysogenic conversion may be detected by the presence of enzyme-encoding genes (such as integrase or ParA/B genes) in the genome [84,217,218]. Moreover, when selecting a phage, their potential to transfer virulent genes or other phage toxic factors between bacteria (transduction) must also be evaluated and taken into consideration [152]. If phages are to be used as therapeutic agents, their safety should be evaluated at the genome level, and phages with lysogenic conversion-related genes (such as integrase or ParA/B genes) or potentially damaging genetic determinants (toxins or antimicrobial resistance genes) should be excluded before further experiments [226,227].

#### 6.1.1. Phages Specificity

A major limitation of phage application is its narrow host range and geographical specificity [39,228,229]. In general, phages are highly specific, infecting only one bacterial genus or even particular strains [224]. Most *Aeromonas* phages showed narrow host specificity, infecting only the original host bacterium [51,93,150,214] or strains of a given bacterial species [91,94,95,149,209,220,230,231] (Table S2). The majority of marine phages are highly host-specific [232,233,234], and about 73% lyse only the original host bacterium [234]. Although phages with a broad host range are hard to find, some examples of virulent phages have shown some promise for therapeutic use in aquaculture, infecting strains of several bacterial species [180,181,217,235,236] and genera [92,237] (Table S2). Liu et al. (2020) reported that the isolated phages had a relatively broad infectivity spectrum against *A. hydrophila* and showed the potential to infect *A. veronii*, *A. caviae* and *A. bestiarum* [236]. In 2011, our research group showed that phage AS-1 infected, beyond the host, *V. anguillarum* and *V. parahaemolyticus* (efficacy of 98.87% and 96.03%, respectively) [237]. In another study, phages AP-1, AP-2, AP-3 and AP-4 showed a broad host range to other genera [92] (Table S2).

Aquaculture environments contain a wide variety of *Aeromonas* sp. that are pathogenic to fish, which hinders the inactivation effects of a single narrow-spectrum phage. Phages that cover a wide spectrum of bacteria are usually in the research area of interest. However, even phages with the broadest host spectrum cannot infect all pathogenic bacterial isolates because of coevolution between bacteria and phage [238]. To overcome this problem, phage cocktails, containing multiple phages that target several possible pathogens, can be used and thus extend their action range [239,240].

#### 6.1.2. Adsorption Rate

Phage adsorption rate may be critical for treatment success. Higher adsorption rates should result in higher phage propagation [241,242]. Storms and colleagues observed that an increase in adsorption efficiency had a similar effect as an increase in the initial MOI; however, the number of phages produced during the amplification phase decreased [243]. Phage AHP-1 adsorption assays showed that approximately 81.5% of phage particles were adsorbed to *A. hydrophila* after 25 min, with an adsorption rate constant of 3.06 × 10^−8^ mL min^−1^ [181]. In other studies, *A. hydrophila* phage adsorption reached over 90% after 40 min [95], while El- Araby et al. (2016) showed a 51% and 66.8% adsorption after 20 and 30 min, respectively [93]. Phage Ahp2 showed a high adsorption efficacy with 96% of phage particles being adsorbed to *A. hydrophila* in the first 18 min [230]. Chen et al. (2018) reported that the five isolated phages (AS-szw, AS-yj, AS-zj, AS-sw and AS-gz) showed strong adsorption to *A. salmonicida* surface and approximately 90% (2.7 × 10^4^ PFU/mL out of 3.1 × 10^4^ PFU/mL) of phage particles adsorbed to *A. salmonicida* within the first 5 min [217]. Nithin et al. (2021) reported that phage AhFM4 showed more adsorption efficacy (96% adsorption within 30 min) when compared with phage AhFM5 (70% of phage was adsorbed within 30 min) [231] (Table S2). That is, phages with a higher adsorption rate should have shorter lysis and vice versa. Because with higher adsorption rates, phages encounter and attack the host more quickly and as such, have a shorter optimal lysis time when compared to phages with a lower adsorption rate.

#### 6.1.3. Latent Period and Burst Size

The selection of phages with a low latent period (time elapsed since phage acid nucleic entry into the cell until the virion progeny are released) and high burst size (number of phage virions produced by each host cell) is very important [32]. A phage is considered a highly lytic phage when it has a short latent period and/or high burst size. Phages shouldn’t be administered in high doses because they cannot diffuse properly. A high burst size not only increases the probability of phage particles reaching the target bacterial cells but also results in a lower risk of selecting phage-resistant bacteria if the phage can eliminate the bacteria faster than they can replicate [240]. Phages with a short latency period are more prevalent during isolation. As such, if they are present in high titer from the beginning, they should be selected for phage therapy because a phage with a long latency period and high burst size may never be found [240]. A phage’s burst size depends mainly on its availability to the bacterial host cells and latent period, being the last affected by phage type, host and environmental conditions [244,245]. Phages with high burst sizes and short latent periods are expected to be more effective in controlling bacteria since these would be able to survive longer in the environment if it maintains their proliferation threshold [246]; however, some phages presenting high burst sizes have been accompanied by long latent periods [166]. Moreover, a rapid increase in phage particles can also contribute to phenomena such as “lysis from without” and other replication anomalies [247]. These can lead to premature cell lysis that hampers phage selection for phage therapy.

An extended latent period can increase phage burst sizes because more time is available for progeny maturation during infection [248]. Few reports, however, have shown high (608 PFU/infected cells) [211] and medium burst sizes (160–316 PFU/infected cells) with the same latent period [236].

*Aeromonas* phages have a wide range of latent periods and burst sizes, going from high burst sizes (608 PFU/infected cells) and short latent periods (15 min) [211] to low burst sizes (5 PFU/infected cells) and long latent periods (40 min) [150].

*A. hydrophila* phages vB-AhyM-AP1 and pAh-1 presented high burst sizes (1413 and 608 PFU/host cell, respectively); however, the latent period of phage vB-AhyM-AP1 (40 min) is higher than that of phage pAh-1 (15 min) [211,235] (Table S2). Phage AhyVDH1 infected *A. hydrophila* producing 274 PFU/host cell [214], TG25P (79 ± 11.9 PFU/host cell) [212]; Akh-2 (139 PFU/host cell) [149] and AHP-1 (97 PFU/host cell) [181]. The latent period of phage AhyVDH1 (50 min) is almost the same as of *A. hydrophila* phages TG25P (40 min) [212], AHP-1 (40 min) [181] and Akh-2 (50 ± 5 min) [149] (Table S2).

Chen et al. (2018) demonstrated that *A. salmonicida* infecting phages AS-szw, AS-yj, AS-zj, AS-sw and AS-gz had a burst size of 145, 98, 86, 86 and 135 PFU/host cell, respectively, with a latent period of approximately 40, 20, 20, 50 and 30 min, respectively [217]. In another study, phage ASP- 1 presented a low burst size of 16 PFU/host cell and a latent period of 30 min [180]. Similar results were obtained with virulent phages AS-A (22 PFU/host cell, 30 min), AS-D (5 PFU/host cell, 40 min) and AS-E (10 PFU/host cell), and that low burst size did not affect the growth reduction of *A. salmonicida* [150].

#### 6.1.4. Phages Stability

To determine the effectiveness of phages, it is necessary to understand their stability under the influence of several parameters [32,150,224,249,250,251,252]. Phage stability may be influenced by several factors like solution composition (presence or absence of particular ions), production process parameters (e.g., temperature, pressure) or environmental conditions (e.g., temperature, pH, salinity and UV radiation) [32,224].

In aquaculture, phages are exposed to the natural variation of environmental conditions, such as temperature, salinity, pH and UV radiation. Some studies showed that phage stability can be negatively affected by those environmental factors [150,249,250,251,252]. These factors can inactivate phage particles by damaging their structural protein elements (capsid, sheath, tail), lipid loss, and/or promoting DNA or RNA structural changes [253]. Phage stability is highly variable, tailed phages are the most stable in adverse conditions and phages with larger capsids have higher survivability [250,253].

Temperature and pH in aquaculture waters are usually moderate and may not influence phage activity; however, the production and formulation process parameters might not be as mild [252]. Therefore, for effective production, it is better to choose phages that are stable at different temperatures and pH.

Temperature is a fundamental factor for virulent phage stability [150,252,254,255,256], playing a crucial role in attachment, genome injection and phage multiplication [250]. At low temperatures, only a small amount of phage particles injects their genetic material into the host cell, as such, few are involved in the amplification phase [257]. On the other hand, the latent period can be prolonged with higher temperatures [257] and degrade the proteins that built up the capsid [224]. Some studies proved that thermal stability is specific for each phage and is different depending on the phage isolate. Phage ASP-1 was stable at high temperatures (4–50 °C) for 1 h [180] but, the viability of phages φZH1 and φZH2 decreased at temperatures above 40 °C [93]. Cheng et al. (2021) reported that phage AhyVDH1 was thermostable at 30 °C and its survival rate decreased to about 66.7% at 40 °C after 60 min, and it was inactivated at 50 °C after 20 min [214]. In another study, Chandrarathna et al. (2020) observed that phage AHP-1 can survive temperatures ranging between 4 °C and 50 °C, even though the infectivity decreased by 75% at 50 °C [181] (Table S2).

Phage stability is also influenced by the acidic and alkaline nature of the environment [250], which affects attachment, infectivity, intracellular replication and phage multiplication [258,259,260]. Adverse pH can inhibit the lysozyme enzyme and the phage protein coat, thus affecting the adherence to the host cell [258]. Due to their protein nature, phage survival usually slowly decreases with environment acidification, promoting their coagulation and precipitation [250]. Generally, the phage lytic activity decreases in pH values ranging between 10 < pH < 5, with optimum pH conditions around neutrality (pH of 6–8) [196,260]. Several phages isolated for *A. salmonicida* and *A. hydrophila* control and presented in Table S2 can tolerate a wide range of pH values, namely phage AS-D (pH 5.5–8) [150], phages AS-yj (pH 5–10) and AS-gz (pH 4–11) [217], phages CT45P and TG25P (pH 5–9) [261], phage ASP-1 (pH 4–11) [180], phage Akh-2 (pH 7–9) [149], phage AhyVDH1 (pH 5–10) [214], phage PVN02 (pH 7–9) [148], phage pAh6.2T (pH 7–11) [94], phage AHP-1 (pH 7–10) [181], phage vB-AhyM-AP1 (pH 5–10) [235], phage pAh-1 (pH 5–11) [211], phages N21 (pH 5–11), W3 (pH 4–10), G65 (pH 4–11), Y71 (pH5–10) and Y81 (pH 4–10) [236], and phages AhFM4 and AhFM5 (pH 5–8) [231].

Phage particles require salts at low concentrations to successfully infect the bacteria and multiply [262]. At low concentrations, salt ions stabilize proteins by neutralizing their charges [256]. At higher concentrations, thermal denaturation of proteins occurs and the structural stability of the phage’s nucleic acid can be affected [263]. *A. salmonicida* phage AS-D remained stable at salinity concentrations of 15%, 20% and 35% for 49 days, after which a decrease of about three orders of magnitude until the 107th day occurred [150]. Phages isolated to control *A. hydrophila* can tolerate a wide range of salinity concentrations, namely phage ASP-1 (0.1–3.5%) [180], phage vB-AhyM-AP1 (0.1–2.0%) [235] and phages AhFM4 and AhFM5 (0.5–2.0%) [231]. Phage pAh6.2TG was relatively stable at a wide range of salinity concentrations (0–40%) for 24 h [94] (Table S2).

UV radiation is one of the main factors that affect phage particle stability in surface coastal waters [150,255,264,265,266,267]. This radiation can degrade phage proteins and form photoproducts such as cyclobutene pyrimidine dimers [268,269], and/or modify their genetic material (either DNA or RNA) [150,264,265,266,267]. Since lethal UV radiation photoproducts are normally thymine dimers, DNA phages are usually more sensitive to damage than RNA phages [256]. Besides, phages with double-stranded genomes are more resistant to UV radiation than single-stranded ones [270,271,272,273,274]. However, our research group observed that AS-D, a double-stranded DNA phage, was able to tolerate UV-B radiation (290–320 nm) while decreasing only by 1.3 log PFU/mL after exposure for 24 h [150]. Phages ΦZH1 and ΦZH2 tolerated UV irradiation, losing 50% of its infectivity after a 100 min and 80 min exposure time, respectively [93].

### 6.2. Multiplicity of Infection (MOI)

The MOI value is an important factor in phage therapy efficiency, it changes depending on the animals, pathogens and phages used in in vivo experiments because of the complex physicochemical environment, host defenses and the outcome in in vitro assays [32,39]. In large-scale production and commercialization of phage products, it would be advantageous and even preferable that phages would be applied in low titres to reduce preparation, purification and application costs [32].

Some authors showed, both under in vitro and in vivo conditions, that the bacterial inactivation occurs in parallel with the MOI or that inactivation occurs earlier with higher MOIs [94,95,180,181,214]. Le et al. (2018) observed that the increase in MOI from 0.01 to 100 promoted a significant increase in the striped catfish survival (*P. hypophthalmus*) [95]. These authors used phage cocktails (phages Φ2 and Φ5) with an MOI of 0.01, 1, and 100 to control *A. hydrophila* infection in striped catfish by injection and obtained relative percent survival of 16.33%, 44.9%, and 100%, respectively. Similar results have been observed for other phages [94]. Treatments with phage pAh6.2TG significantly improved Nile tilapia survival when exposed to lethal doses of *A. hydrophila*, with relative percent survival of 73.3% and 50% for MOIs of 1.0 and 0.1, respectively [94]. Cheng et al. (2021) reported that a higher phage dosage (MOI of 10) had a higher effect on *A. hydrophila* reduction [214]. Similar results were noted by other researchers for phages ASP-1 and AHP-1 against *A. salmonicida* and *A. hydrophila* strain, respectively [180,181]. However, Chen et al. (2018) observed that phage-resistant bacterial variants may be induced more rapidly by heavy phage concentrations than those treated with lower concentrations. These authors showed that bacterial density (OD600) rapidly increased when incubated with higher MOIs (10 and 1), even though bacterial inactivation occurred earlier than at lower MOIs (0.1 and 0.01) [217]. Similar results were obtained by Jun et al. (2013) for phage pAh6-C against *A. hydrophila* [209].

### 6.3. Administration Routes

The route and time of application are other factors that influence phage therapy efficiency. In aquaculture, phage delivery can be done through immersion, injection, within the feed, and topical application. The phage-impregnated feed is more appropriate in prophylactic efforts as infected fish may not consume their food [275]. The selection of the right application method is essential; however, each biological system is different and should be considered independently [224]. Whatever the application method, it is important that the phages particles contact the bacterial host, either in water, on the surface or inside fish [275]. This can be easily achieved in fish tanks by maintaining water circulation through pumps or extensive fish mobility in high-stocking tanks [275].

Immersion in seawater tanks is the most common technique used in phage therapy studies to control *Aeromonas* sp. in fish [51,91,92,93,94,149,209,218,219,276] (Table S3). Treating loach (*M. anguillicaudatus*), rainbow trout (*O. mykiss*), Nile tilapia (*O. niloticus*), brook trout (*S. fontinalis*) and Senegalese sole (*S. senegalensis*) by immersion could provide significant protective effects against *A. hydrophila* infection [51,91,92,93,94,149,209,218,276]. However, due to *A. salmonicida* subsp *salmonicida* high infectivity, even at extremely low concentrations, in Atlantic salmons, phage treatment by immersion was ineffective in preventing or treating the pathogen [219].

Some studies reported phage protective effects against *A. hydrophila* and *A. salmonicida* infection by intraperitoneal injection [91,95,209,211,219,220,277] (Table S3). Le et al. (2018) reported that intraperitoneal injections in catfish provide significant protective effects, with their survival rate increasing when the MOI value increased [95]. In another study, the administration of phage PAS-1 in a rainbow trout model infected with *A. salmonicida* subsp. *salmonicida* showed notable protective effects, with increased survival rates and delayed death by almost 1 day [220]. A dissimilar result was achieved by Verner–Jeffreys et al. (2007), who showed that no protection was offered by intraperitoneal injection of phages O, R and B, compared to the positive challenge group [219]. The labour-intensive and time-consuming administration of phages by intraperitoneal injections can constitute a disadvantage for fish treatment in catfish farms.

Some reports compare different methods of phage administration. Jun et al. (2013) evaluated the protective effects of intraperitoneal injection and oral administration of phages pAh1-C and pAh6-C against *A. hydrophila* [209]. In this study, the fish were infected with two different bacterial concentrations (10^6^ CFU/fish and 10^7^ CFU/fish) and treated with phages phAh1-C and phAh6-C (10^7^ PFU/fish). The fish treated with phages by intraperitoneal injection and oral administration showed lower mortality rates than the control group. In fish infected with 10^6^ CFU/mL of *A. hydrophila* and treated by intraperitoneal injection, no mortality was observed in the groups treated with phages pAh1-C or pAh6-C over 7 days (cumulative mortalities in the control group was 39.17 ± 3.82%). However, when the fish were infected with 10^7^ CFU/mL, the cumulative mortality was 43.33 ± 2.89% for phage pAh1-C and 16.67 ± 3.82% for phage pAh6-C (cumulative mortality in the control group was 100%) [209]. When the fish were infected with 10^6^ and 10^7^ CFU/mL of *A. hydrophila* and fed with phage-coated food the cumulative mortalities were 17.50 ± 2.50% and 46.67 ± 3.82% for phage pAh1-C and 11.67 ± 3.82% and 26.67 ± 2.89% for phage pAh6-C. The cumulative mortalities in the control group were 38.33 ± 2.50%; 2nd trial, 95.83 ± 3.82% [209]. Cao et al., in 2016, published a report in which they described the use of phage MJG to control *A. hydrophila* in rainbow trout (*O. mykiss*) [91]. The fish were infected with *A. hydrophila *(10^8^ CFU/fish) and treated with a single dose of phage MJG (3.2 × 10^6^ PFU/fish) administered intraperitoneally 2 h post-bacterial infection or immersed in water for 15 min with phage at a concentration of 3.2 × 10^6^ or 3.2 × 10^5^ PFU/mL. Phage MJG injection completely protected the fish from *A. hydrophila* infection (cumulative survival in the control was 40%). In the immersion treatment, the cumulative survival was 100% and 80% with phage concentrations of 3.2 × 10^6^ PFU/mL and 3.2 × 10^5^ PFU/mL, respectively [91].

Another very important factor for the success of phage therapy is the time of administration. This parameter is highly dependent on the type of disease and how advanced the infection is. Verner-Jeffreys et al. (2007) observed that Atlantic salmon injected with the phage cocktail (phages O, R and B), immediately after bacterial inoculation, died at a significantly slower rate than those without phage treatment or treated 24 h after inoculation [219].

A phage’s ability to cross the epithelial barrier or withstand gastric conditions determine how it can be administrated. These parameters may impact phage degradation in the gastric tract and may decrease phage therapy effectiveness [278]. To avoid these problems, coatings can be applied to fish feed containing phages [279]. The edible whey protein isolate coatings loaded with phages enhance fish treatment by reducing phage activity loss. Results from a simulation assay for gastric-intestinal digestion showed that this method enhances phages stability and reduces bacterial levels. Furthermore, it allows to control phage release in saltwater and protects them until they reach their destination [39,278].

### 6.4. Bacterial Resistance

Phage-resistant bacteria is probably the major concern regarding phage therapy, which could jeopardize favorable treatment outcomes. During treatment, some mutations will spontaneously occur and phage-resistant bacteria will regrow. However, most of these experiments are performed in a nutrient-rich medium without the presence of competition [150,166]. In these studies, since the remaining viable bacteria are not challenged by the host’s immune system and the culture conditions are suitable for bacterial growth, resistant bacteria can regrow to concentrations similar to those of the non-treated control [150,166]. Our research group obtained similar results in vitro; however, the same didn’t happen in vivo, where the phage-resistant bacterial mutants were at lower concentrations than the susceptible bacterial population [51].

Phage-resistant bacteria can develop from (i) alteration or loss of bacterial cell surface receptors; (ii) receptor(s) blockage by the bacterial extracellular (exopolysaccharide) matrix; (iii) inhibition of phage DNA penetration; (iv) production of modified restriction endonuclease enzymes that effectively hydrolyze phage DNA; or (v) inhibition of intracellular phage assembly [280]. Of these, changes in bacterial cell surface phage receptors represent the most frequent cause of resistance [280,281].

The use of phage cocktails during phage treatment may help overcome the problem of bacterial phage resistance. However, its success requires phages that do not have overlapping cross-resistance, i.e., bacterial mutants resistant to one phage but still sensitive to the other, and vice versa [39], using, for instance, phages from different families. Therefore, cocktails made of phages that only target bacterial lipopolysaccharides, for example, should be less successful than cocktails containing phages that target different receptors. Furthermore, the different phages should be able to be adsorbed onto the bacterial cell surface and have their genome injected. On the other hand, the different phages may interfere with each other upon co-infection, which is problematic [246]. However, phage cocktails do not prevent the emergence of phage-resistant mutants [150,166,212], although they can limit the development of resistant bacteria [166]. In one study, the frequency of *A. hydrophila* mutants resistant to phage AH-1 (3.10 × 10^−3^) and AH-4 (1.14 × 10^−3^) was higher than that observed with the phage cocktails (8.26 × 10^−4^) [166]. However, in another study, the rate of phage-resistant bacteria to the phage AS-D (9.11 × 10^−5^) was slightly lower than that observed with phage cocktails AS-A/AS-D (1.64 × 10^−5^), AS-D/AS-E (1.05 × 10^−4^) and AS-A/AS-D/AS-E (1.70 × 10^−4^) [150]. As such, the combination of different therapeutic approaches should be considered to prevent and combat the emergence of microbial resistance. The combined treatment with phage AHP-1 and chloramphenicol (5 μg mL^−1^) was more promising than individual treatments [181].

Several researchers have said that a small frequency of resistant mutants is not problematic and should not hamper phage application against pathogenic bacteria [282,283], and other authors stated that even bacterial exposure to phages could result in a “fitness cost” for the bacteria [284,285] and contribute to their faster elimination from the environment when compared to their wild-type counterparts. In fact, our research group observed that colonies of phage-resistant bacteria were smaller and took several more days of incubation (5–6 days) to grow than the non-resistant bacterial colonies (24 h) [51]. These results indicate that phage-resistant bacteria tend to be less fit and, consequently, are expected to be eliminated from the environment more rapidly than their wild-type counterparts. However, this may vary across environments and the competition level for resources [286,287].

The specialty literature has stated that the phage itself can overcome the host’s resistance through co-evolution [288,289] and our research group observed that after successive streak-plating steps, phage-resistant bacteria also mutated [290], with positive spot tests occurring only after the fourth and fifth steps. These authors confirmed these results by Fourier-Transform Infrared Spectroscopy (FTIR), where the spectra obtained from the fourth and fifth streak-plating colonies were similar to the ones from phage-sensitive control colonies, suggesting that these colonies are more similar to the control phage-sensitive bacteria than the colonies from streak-plating steps one, two and three [290].

### 6.5. Immune Response

A concern that hampers the success of phage therapy is the immunogenicity of phage particles. Phages can stimulate an immune response in fish, causing both specific or adaptative and non-specific or innate responses. The innate immune system reacts first by producing phagocytes to remove phages. The adaptive immune system enhances the first response with lymphocytes and antibodies. These systems work together, preventing viral attachment to bacteria, which can reduce or halt the therapeutic effect [249,291].

The existence of phage neutralizing antibodies before starting therapy or after repeated therapeutic administration might be a reason for phage therapy’s failure [292]. High-titer phages usually stimulate the immune system of immunocompetent hosts [292]. However, antibody production depends on the route of phage administration, application timing and dosage, and the phage individual features [293]. The administration of *A. salmonicida* phage PAS-1 (MOI of 10000), in rainbow trout, showed significant neutralizing properties of its sera 10 days and 15 days after intramuscular administration which declined after 30 days [220]. However, this neutralization wasn’t due to the presence of phage particles in the kidneys and occurred after the phage had been removed [220].

In a recent study, the immunogenicity of phages is highlighted as a profitable aspect. The authors used a phage lysate, composed of inactivated lytic phage pAh 6-c antigens to develop a vaccine for the prevention of *A. hydrophila* infection in *C. carpio*. Furthermore, to increase the effectiveness of the vaccine, the authors also tested the encapsulation of phage lysate and formalin-killed cells of *A. hydrophila* JUNAH strain with poly(lactic-co-glycolic acid) (PLGA) at low or high concentrations for intraperitoneal injection in fish [294]. Groups vaccinated with high doses of phage lysate antigen obtained higher agglutination concentrations than all other groups at 4-weeks and 6-weeks post-vaccination. Fish immunised with phage lysate vaccines had a higher survival rate than fish immunised with the formalin-killed cells vaccine. Vaccines with the phage lysate antigen also resulted in higher IL-1β and lysozyme C gene expression 7-days and 2-weeks post-vaccination, and higher TNF-α gene expression was seen 7-days post-vaccination. These results suggest that phage lysate antigen may induce stronger immune responses than formalin-killed cells-based vaccines and can be more effective as a novel inactivated antigen to prevent *A. hydrophila* infection in *C. carpio* [294]. Previous studies speculated that the phage’s surface proteins can be recognized as foreign antigens by the host immune system and trigger stimulating immune responses. Moreover, it has been reported that, due to bacterial endotoxins such as lipopolysaccharides (LPS) and bacteria lysis remains, which may be in phage preparations or be produced by sudden lysis of many bacterial cells, an acute immune response can be induced [295]. In addition, serum TNF-α levels and the production of TNF-α and IL-6 by blood cells can be normalized by effective phage therapy [296]. However, continuous exposure to the same phage can provoke adaptive immune responses with antibody production, which hampers the effectiveness of phage treatment [297].

Another challenge concerning phage therapy and the immune system response is the difficulty in reaching the site of infection in in vivo conditions. For phage therapy to be efficient, the amount of phage must be sufficient to reach the target bacterial cells [298]. According to Kalatzis et al. (2018), phage therapy in fish can make the adaptive immune system respond by eliminating phages from the body and preventing them from reaching the infection site [299]. The possible solution to overcome this issue is to study each case and choose carefully how to administer, the dosage, buffers and phage exposure time [300]. To protect phages when they enter the fish system, different approaches can be considered namely: phage microencapsulation, use of protective agents or appropriate buffers [249]. Screening phage mutants by genetic or chemical methods can also be used to reduce the immunogenicity of the surface proteins and thus prevent phages from being eliminated by the fish immune system so easily [301]. Furthermore, phage cocktails composed of different phages are desirable because they could help phage survival in living systems by neutralizing antibodies [249].

Phages possess strong immunomodulating and anti-inflammatory properties. The possible mechanisms responsible for these effects may involve LPS binding, inhibition of excessive production of reactive oxygen species and induction of IL-10 production [302]. Schulz et al. (2019a) studied the immunomodulatory activity of the commercially available phage cocktail designated BAFADOR^®^, a phage preparation against *A. hydrophila* and *Pseudomonas fluorescens*, in rainbow trout (*O. mykiss*), when a mixed infection of *Aeromonas* and *Pseudomonas* was induced [303]. For this, the authors determined the proliferative response of pronephros lymphocytes after stimulation with LPS or concanavalin A, as well as metabolic activity and potentially lethal activity of spleen phagocytes, total protein and total Ig contents, lysozyme and ceruloplasmin activities. Besides obvious antibacterial action against *A. hydrophila* and *P. fluorescens*, which decreased the mortality of rainbow trout, it also elevated immunoglobulin, lysozyme and protein levels, along with an increase in spleen phagocytes activity and pronephros lymphocytes proliferation [303]. The same group also studied the effect of BAFADOR^®^ on the European eel (*A. anguilla)* immune system when a mixed infection of *Aeromonas* and *Pseudomonas* was induced [304]. Similar to the previous study, the results showed that BAFADOR^®^ is well tolerated by the fish organism stimulating the parameters of cellular and humoral immunity and reducing the mortality of European eels after experimental challenge [304]. Cao et al. (2020) reported that the pro-inflammatory cytokines expression levels (IL-8 and IL-1β) were significantly higher in the spleen of phage MJG-treated fish than in PBS-treated fish 1- or 2-days post-infection but significantly lower in fish treated with PBS 3-days post-infection [91]. Therefore, phage treatment seemed to stimulate an early strong inflammatory response that weakened over time [91]. The proper inflammatory response removes harmful stimuli and restores health, but excessive and uncontrolled inflammation may damage the healthy tissues [305]. The strong inflammatory response may be associated with the release of endotoxins after cell lysis by phage MJG [91]. However, inflammation enhancement by phage treatment was significantly weaker than that by PBS treatment 3-days post-infection [91]. This may be explained by the low bacterial concentration and anti-inflammatory abilities of phage MJG [91]. Consistent with this result, phage MJG successfully restored tissue damage and eliminated any clinical signs of *A. hydrophila* infection in the fish [91]. In another study, Chandrarathna and co-workers verified that the immune gene expression of zebrafish upon continuous bath exposure to phage AHP-1 was significantly high (il-6 and sod-1) or slight (tnf-α, il1-β, il-10, and cxcl-8a) than the controls at the beginning of phage exposure. However, those values lowered to minimum levels 12 days after post-phage exposure, suggesting no adverse immune responses had occurred for the phage AHP-1 dose used, and potential for phage therapy [181].

### 6.6. Phage’s Environmental Influence

Virulent phages are usually highly specific to a single species or even strain of bacteria and therefore, presumably, cause much less damage to the natural non-target bacterial communities and the normal intestinal fish flora. Though, as non-pathogenic bacteria have an important ecological role in aquatic systems, such as aquaculture systems, the effects of phage infection on bacterial communities in aquaculture water must be evaluated before applying phage therapy.

Phage therapy in the aquaculture system may have an impact on the environment by disrupting the microbiome [224]. Phages regulate the number of certain bacteria in a given environment and consequently change the bacterial proportions in that community [224]. Phages also have an important impact on the global biosphere organic matter cycle by releasing organic compounds through bacterial cell lysis [306]. Knowledge about these factors is especially important in the aquatic environment because it allows for rapid dissemination and acts as a vector for phages [39].

The likelihood of disruption to environmental bacterial communities can be reduced by using smaller phage doses. However, if phages are introduced in a small quantity, their concentration might be ineffective to control the pathogenic bacteria [39,224]. On the other hand, phages can reproduce and spread in the environment, not just in the targeted aquaculture system [239,307]. Despite being harmless, it is important to test their impact on the treated microbial community before any industrial-scale application.

Our research group evaluated the impact of phage AS-1 (*A. salmonicida* phage) on the bacterial community structure of an aquaculture system and observed a moderate impact on the overall bacterial community despite a broad host range [237]. In 2016, we also reported the impact of phage AS-A on natural bacterial communities of an aquaculture system and bacterial community associated with fish intestinal tract [51]. We observed that the addition of phage AS-A to the aquaculture water only significantly affected the bacterial community of the fish’s intestinal tract and not the natural structure of the bacterial community [51].

## 7. Conclusions and Future Perspectives

Currently, the effectiveness of antibiotics is faltering as more and more antibiotic-resistant strains are identified. As such, alternative treatments such as phage therapy should be explored. Most of the studies reviewed in this paper showed the effect phages have in the control of *Aeromonas* species on fish, thus providing a positive outlook on the future benefits of this technology to treat aquaculture diseases. However, the existing studies are restricted to two *Aeromonas* species, *A. salmonicida* and *A. hydrophila*. Therefore, more studies are needed to optimize phage application under field conditions and to better understand the interactions between host fish, bacteria and phage.

The ability of phages to control *Aeromonas* species in aquaculture systems depends on several factors, such as phage selection, MOI, environmental factors that affect lytic phage viability (e.g., temperature, salinity, pH, UV radiation), administration routes and bacterial resistance to phages. In addition, the data obtained in in vitro assays cannot be directly applied to in vivo assays, nor can in vivo data for one phage be extrapolated to another phage. Before applying this approach commercially, phages must undergo efficacy testing to demonstrate their effectiveness and safety. Several factors need to be standardized and taken into account such as cost-effectiveness, administration method, the MOI that produces the best bacterial inactivation and stability of phage preparations. It is also necessary to explore the potential impact on the natural bacterial community and fish health, as a function of the type of bacteria and different environmental conditions, to allow its integration as a new antimicrobial processing technology in aquaculture.

Phage therapy is cost-effective, eco-friendly, safe for aquaculture species and end-users such as humans and animals. Despite the development of some bacterial degree of resistance towards phages, the harmful effects are negligible compared to the development of antibiotic resistance. The predisposition of bacteria to develop resistance to phages is ten times slower than that of antibiotics and bacteria resistant to one phage can be infected by other phages with similar target ranges.

## Figures and Tables

**Table 2:** Companies involved in the development of phage-based products to control or prevent bacterial infections in aquaculture.

Company	Country	Target Application	References
Intralytix Inc.	Baltimore, MD, USA	Phage-based application to fight *V. coralliilyticus* and *V. tubiashii* infections in hatchery-raised oysters	[203]
BASF New Business GmbH	Ludwigshafen, Germany	Products that mix phages covalently to particles into the feed to treat infections caused by *Vibrio*, *Yersinia*, *Aeromonas, Rickettsia*, *Moritella*, *Lactococcus*, *Piscirickettsia*, *Flavobacterium, Pseudomonas,* or *Photobacterium*	[204]
Proteon Pharmaceuticals S.A.	Łódz, Poland	Natural feed additive called BAFADOR^®^ that can control bacterial infections caused by *Pseudomonas* spp and *Aeromonas* spp. serotypes in commercial aquaculture	[50]
Fixed Phage Ltd.	Glasgow, Scotland	Phage particles immobilized in pellets that can be added to fish and crustacean feed to treat bacterial infections in aquaculture, including Early Mortality Syndrome in shrimps and *Flavobacteria* infections in salmonids	[204]
ACD Pharma	Oslo, Norway	Phage-based solutions against several aquaculture pathogens; CUSTUS^®^ YRS is a product that reduces the infective pressure from *Y. ruckeri* in aquaculture water	[205]
Mangalore Biotech Laboratory	Karnataka, India	A product called LUMI-NIL MBL prevents and treats *Vibrio harveyi*-caused luminous vibriosis	[206]
Phage Biotech Ltd.	Rehovot, Israel	Phage treatment for *V. harveyi* in aquaculture shrimps	[202]
Biologix	Australia	Phage treatment for *Vibrio* sp. associated with mortalities in aquaculture	[207]
Aquatic Biologicals	Greece	Phage treatment against several pathogens associated with mortalities in aquaculture	[208]

## Data Availability

Not applicable.

## Supplementary Materials

The following supporting information can be downloaded at: https://www.mdpi.com/article/10.3390/antibiotics11020163/s1. Table S1: *Aeromonas* species typically associated with fish diseases and clinical signs; Table S2: In vitro studies of phage therapy for controlling *Aeromonas* sp. in fish; Table S3: In vivo studies of phage therapy for controlling *Aeromonas* sp. in fish. References [307,308,309,310,311,312,313,314,315,316,317,318,319,320,321,322,323,324,325,326,327,328,329,330,331,332,333,334,335,336,337,338,339,340,341,342,343,344,345,346,347,348,349,350,351,352,353,354,355,356,357,358,359,360,361,362,363,364,365,366,367,368,369,370,371,372,373,374,375,376,377,378,379,380,381,382,383,384,385,386,387,388,389,390,391,392,393,394] are cited in the supplementary materials.
