# Supplementary material for: Bacteriophages in the Control of Aeromonas sp. in Aquaculture Systems: An Integrative View
Source: Antibiotics (Basel). 2022 Jan 27;11(2):163. doi: 10.3390/antibiotics11020163 (PMC8868336; doi:10.3390/antibiotics11020163)
Supplement: Supplementary file 1 [file antibiotics-11-00163-s001.zip › antibiotics-1506288-supplementary.pdf]

Supplementary Materials

Table S1. *Aeromonas* species typically associated with fish diseases and clinical signs.

***Aeromonas* species**	**Fish species**	**Clinical symptoms**	**References**
Aeromonas salmonicida	*Salmo truta*<br>*Oncorhynchus mykiss*<br>*Salvelinus fontinalis*<br>*Salmo labrax*<br>*Salmo salar*<br>*Salmo macrostigma*<br>*Thymallus thymallus*<br>*Clarias gariepinus*<br>*Dicentrarchus labrax*<br>*Salvelinus willoughbii*<br>*Salvelinus alpinus*<br>*Cyprinus carpio*<br>*Carassius gibelio*<br>*Cyprinus carpio koi*<br>*Oreochromis mossambicus*<br>*Scophthalmus maximus*<br>*Perca fluviatilis*<br>*Siniperca chuatsi*<br>*Coreius guichenoti*<br>*Rutilus rutilus*<br>*Anarhichas minor*<br>*Anarhichas lupus*<br>*Anguilla anguilla*<br>*Gadus morhua*	Behavioural: Moribund behaviour.<br><br>Observable manifestations: Furunculosis (swelling and ulcerations in the dorsal muscles, blood clots in the back and a reddened and swollen anus), skin ulceration with sunken central areas, muscle fibre necrosis, gill proliferation, haemorrhages on the skin and on the base of the fins, fin erosion, eye injuries and haemorrhages on the eyes, opaque lens, inflammation in the ventral part of the operculum, skin erosion, pale gills. Skin nodules convex and with skin swelling (2-6mm), in some cases the nodules were grey to reddish delineated by pale tissue and may undergo erosion.<br><br>Internal observations: Swelling of the spleen and kidney, pale liver, petechiae, haemorrhages on the stomach, gills, spleen, liver and intestine and excessive secretion of mucus in the gastrointestinal tract, septicaemia and anus relapse.<br><br>In some cases, no clinical symptoms were observed prior to autopsy, in others, there was no visible internal organ damage.	[81, 308-328]
Aeromonas hydrophila	*Salmo trutta*<br>*Oncorhynchus mykiss*<br>*Oncorhynchus masou*<br>*Salvelinus fontinalis*<br>*Salmo labrax*<br>*Salmo macrostigma*<br>*Oryzias latipes*<br>*Tachysurus fulvidraco*	Behavioural: Anorexia, loss of appetite, moribund behaviour, lethargy, air gulping and swimming abnormalities: swimming at the surface of the pond, slow swimming, weak swimming, swimming erratic and loop swimming.<br><br>Observable manifestations: Skin redding, fish with apparent lighter colour, shallow to deep skin ulceration with white rings, muscle fibre and tissue necrosis, gill proliferation, external skin haemorrhage and on the base of the anal fin, fin erosion, eye injuries, exophthalmia, pale gills and distended abdomen (dropsy).	[13, 67, 68, 73, 87, 88, 101, 102, 121, 143, 314-316, 320, 326, 328-370]

	Clarias gariepinus<br>*Heteobranchus longifilis*<br>*Ictalurus punctatus*<br>*Pangasianodon hypophthalmus*<br>*Clarias gariepinus*<br>*Ictalurus furcatus*<br>*Ictalurus punctatus*<br>*Dicentrarchus labrax*<br>*Ictiobus cyprinellus*<br>*Monopterus albus*<br>*Megalobrama amblycephala*<br>*Sparus aurata*<br>*Trachurus mediterraneus*<br>*Salvelinus alpinus*<br>*Cyprinus carpio*<br>*Ctenopharyngodon idella*<br>*Hypophthalmichthys molitrix*<br>*Aristichthys nobilis*<br>*Carassius auratus*<br>*Carassius gibelio*<br>*Cirrhinus mrigala*<br>*Cirrhinus cirrhosus*<br>*Cyprinus koi*<br>*Labeo rohita*<br>*Labeo catla*<br>*Oreochromis niloticus*<br>*Oreochromis mossambicus*<br>*Acipenser baerii*<br>*Acipenser gueldenstaedtii*<br>*Acipenser stellatus*<br>*Huso huso*<br>*Acipenser ruthenus*<br>*Prochilodus lineatus*<br>*Poecilia reticulata*<br>*Poecilia latipinna*<br>*Scophthalmus maximus*<br>*Etroplus suratensis*<br>*Myxocyprinus asiaticus*<br>*Perca fluviatilis*<br>*Astacus leptodactylus*	Internal observations: Haemorrhagic septicaemias with haemorrhage in internal organs, intestine and a red tinged ascitic fluid, hepatopancreatic shrinking, enlarged bladder, swelling of the spleen and kidneys, pale or green liver, petechiae, yellow fluid filled abdomen and distended intestine.<br><br>In some cases, no clinical symptoms were observed prior to autopsy, in others, there was no visible internal organ damage.	

	Heros severus<br>*Herichthys cyanoguttatus*<br>*Hoplarchus psittacus*<br>*Xiphophorus clemenciae*<br>*Xiphophoporus helleri*<br>*Xiphophorus maculatus*<br>*Phenacogrammus interruptus*<br>*Maccullochella peelii*<br>*Litopenaeus vannamei*<br>*Thunnus atlanticus*<br>*Colossoma macropomum*<br>*Piaractus mesopotamicus*<br>*Piaractus brachypomus*<br>*Potamonautes sidneyi*<br>*Pseudoplatystoma corruscans*<br>*Pseudoplatystoma fasciatum*		
Aeromonas caviae	*Salmo trutta*<br>*Oncorhynchus mykiss*<br>*Salmo labrax*<br>*Salmo macrostigma*<br>*Clarias batrachus*<br>*Clarias gariepinus*<br>*Dicentrarchus labrax*<br>*Cyprinus carpio*<br>*Carassius gibelio*<br>*Cyprinus koi*<br>*Labeo rohita*<br>*Labeo catla*<br>*Oreochromis mossambicus*<br>*Acipenser baerii*<br>*Acipenser gueldenstaedtii*<br>*Acipenser ruthenus*<br>*Prochilodus lineatus*<br>*Poecilia reticulata*<br>*Perca fluviatilis*	Behavioural: Lethargy, imbalance swimming and loss of equilibrium and ventilating.<br><br>Observable manifestations: Skin ulceration with red ulcers displaying a white ring, tissue and muscle fibre necrosis, gill proliferation, haemorrhages in the body and in the base of the fins, fin erosion, eye injuries, abdominal swelling, gill inflammation, pale gill tissue and distended abdomens (dropsy).<br><br>Internal observations: Pale liver, enlarged spleen, internal haemorrhages and excess of fluids.<br><br>In some cases, no clinical symptoms were observed prior to autopsy, in others, there was no visible internal organ damage.	[314, 316, 320, 326, 330, 331, 334, 338, 348, 353, 360, 365, 371, 372]
Aeromonas sobria	*Salmo trutta*<br>*Oncorhynchus mykiss*	Behavioural:<br>Lethargy, imbalance swimming and loss of equilibrium and ventilating.	

	Oncorhynchus masou<br>*Salmo labrax*<br>*Salmo macrostigma*<br>*Oryzias latipes*<br>*Clarias gariepinus*<br>*Rhamdia quelen*<br>*Cyprinus carpio*<br>*Ctenopharyngodon idella*<br>*Hypophthalmichthys molitrix*<br>*Carassius auratus*<br>*Carassius gibelio*<br>*Cyprinus koi*<br>*Oreochromis mossambicus*<br>*Acipenser baerii*<br>*Acipenser gueldenstaedtii*<br>*Poecilia reticulata*<br>*Poecilia latipinna*<br>*Perca fluviatilis*<br>*Heros severus*<br>*Herichthys cyanoguttatus*<br>*Xiphophorus clemenciae*<br>*Xiphophoporus helleri*<br>*Xiphophorus maculatus*<br>*Phenacogrammus interruptus*<br>*Misgurnus mizolepis*	Observable manifestations: Skin ulceration with red ulcers displaying a white ring, tissue and muscle fibre necrosis, gill proliferation, haemorrhages in the body and in the base of the fins, fin erosion, eye injuries, abdominal swelling, gill inflammation, pale gill tissue and distended abdomens (dropsy).<br><br>Internal observations: Pale liver, enlarged spleen, internal haemorrhages and excess of fluids.<br><br>In some cases, no clinical symptoms were observed prior to autopsy, in others, there was no visible internal organ damage.	[11, 15, 102, 314, 316, 326, 331, 338, 339, 344, 348, 351, 358, 359, 365, 372, 373]
Aeromonas bestiarum	*Salmo trutta*<br>*Oncorhynchus mykiss*<br>*Salmo labrax*<br>*Salmo macrostigma*<br>*Clarias gariepinus*<br>*Cyprinus carpio*<br>*Ctenopharyngodon idella*<br>*Hypophthalmichthys molitrix*<br>*Carassius auratus*<br>*Cyprinus carpio koi*<br>*Oreochromis mossambicus*<br>*Acipenser baerii*<br>*Acipenser gueldenstaedtii*	Behavioural: Moribund, unbalanced swimming, loss of appetite<br><br>Observable manifestations: Skin ulcers, external haemorrhages and fin rot.<br><br>Internal observations: Haemorrhages<br><br>In some cases, no clinical symptoms were observed prior to autopsy, in others, there was no visible internal organ damage.	[314, 315, 326, 344, 353, 362]

	Acipenser stellatus<br>*Huso huso*<br>*Acipenser ruthenus*<br>*Prochilodus lineatus*<br>*Astacus leptodactylus*<br>*Heros severus*<br>*Herichthys cyanoguttatus*<br>*Hoplarchus psittacus*		
Aeromonas veronii	*Salmo trutta*<br>*Oncorhynchus mykiss*<br>*Oncorhynchus masou*<br>*Salmo labrax*<br>*Salmo macrostigma*<br>*Oryzias latipes*<br>*Ictalurus punctatus*<br>*Clarias gariepinus*<br>*Ictalurus furcatus*<br>*Dicentrarchus labrax*<br>*Monopterus albus*<br>*Cyprinus carpio*<br>*Ctenopharyngodon idella*<br>*Hypophthalmichthys molitrix*<br>*Carassius auratus*<br>*Carassius gibelio*<br>*Cyprinus koi*<br>*Labeo rohita*<br>*Oreochromis niloticus*<br>*Oreochromis mossambicus*<br>*Oreochromis niloticus*<br>*Acipenser baerii*<br>*Acipenser gueldenstaedtii*<br>*Acipenser stellatus*<br>*Huso huso*<br>*Lota lota maculosa*<br>*Prochilodus lineatus*<br>*Streaked prochilodus*<br>*Poecilia latipinna*<br>*Silurus glanis*	Behavioural: Moribund, loss of appetite, anorexia, lethargy and ventilating, slow swimming, unbalanced swimming and erratic swimming.<br><br>Observable manifestations: Skin redding or darkening, ulcers, eye swelling, haemorrhages of the operculum, mouth, fin base and body, icteric appearance, reduced growth, abdominal distention, ascites, rotting and eroded fins, swelling of the cloaca, pale, pink or grey gills, exophthalmia, gill necrosis, eye haemorrhages, swollen head and detached scales.<br><br>Internal observations: Petechial haemorrhages, internal haemorrhages, fluid on internal cavities, nodules in the kidney and spleen, enlarged organs, liver necrosis, haemorrhages in the spleen and liver.<br><br>In some cases, no clinical symptoms were observed prior to autopsy, in others, there was no visible internal organ damage.	[13, 14, 143, 183, 314, 315, 318, 326, 339, 344, 348, 353, 359, 362, 372, 374-392]

	Myxocyprinus asiaticus<br>*Astacus leptodactylus*<br>*Heros severus*<br>*Herichthys cyanoguttatus*<br>*Hoplarchus psittacus*<br>*Xiphophorus clemenciae*<br>*Astronotus ocellatus*<br>*Phenacogrammus interruptus*<br>*Danio rerio*		
Aeromonas jandaei	*Oncorhynchus mykiss*<br>*Pangasianodon hypophthalmus*<br>*Clarias gariepinus*<br>*Cyprinus carpio*<br>*Ctenopharyngodon idella*<br>*Hypophthalmichthys molitrix*<br>*Carassius auratus*<br>*Carassius gibelio*<br>*Cyprinus koi*<br>*Labeo rohita*<br>*Oreochromis niloticus*<br>*Oreochromis mossambicus*<br>*Acipenser baerii*<br>*Acipenser stellatus*<br>*Huso huso*<br>*Etroplus suratensis*<br>*Astacus leptodactylus*<br>*Heros severus*<br>*Herichthys cyanoguttatus*<br>*Hoplarchus psittacus*	Behavioural: Moribund, unbalanced swimming, lethargy and loss of appetite.<br><br>Observable manifestations: Necrosis, haemorrhaging and reddish lesions, skin ulcers, tail and fin rot.<br><br>Internal observations: Haemorrhages, brain and intestine with blood congestion.	[14, 314, 344, 345, 348, 357, 362, 383]

Table S2. In vitro studies of phage therapy for controlling *Aeromonas* sp. in fish.

Bacterial strain	Phage	Phage origin	Taxonomy	Infectivity/host range	Survival of the phage	Phage genome size (kb)	Latent period (min)	Burst size (PFU/infected cell)	Outcome	Reference
Aeromonas hydrophila	Phage AH1	Isolated from pond and sewage waters.	Caudovirales	n.a	Phage AH1 was stable in the different water sources (fish pond waters, sterilized tap water and distilled water) tested for 4 days.	n.a.	40	160	Phage reduced *A. hydrophila* concentration by 6 log CFU/mL after 2 h of incubation when compared with the initial concentration ($2x10^7$ cells/mL).	[174]
Aeromonas salmonicida	Phages A, B, C, D, E, F, G, H, I, J, K, L, M, N, O, P, Q and R	Phages A, B, F, G, H, I, J, K and L were isolated from a fish farm effluent (UK).<br><br>Phage C was isolated from a reservoir (UK).<br><br>Phages D and E were isolated from a fish farm effluent in Australia and Canada, respectively.<br><br>Phages M, N and O were isolated from a fish farm effluent (France).<br><br>Phages P, Q and R were isolated from a reservoir (France).	Caudovirales	Lysed some *A. salmonicida* strains.	n.a.	n.a.	n.a.	n.a	n.a	[216]

Aeromonas hydrophila	Several phages of *A. hydrophila*	Isolated from water samples in southern Taiwan.	n.a	n.a	n.a	n.a	n.a	n.a	When *A. hydrophila* was added to the pond water (6 × 10^5/ml) the number decrease 250-fold at an MOI of 0.23 in 8 h.	[393]
Aeromonas salmonicida	Phage AS-1	Isolated from water samples of the aquaculture Corte Freiras, Ria Aveiro, Portugal.	n.a	Phage AS-1 infected, beyond the host, *Vibrio anguillarum* and *Vibrio parahaemolyticus* (efficacy of 98.87 and 96.03 %, respectively).	Phage survived in seawater for 91 days. at 25 °C.	n.a.	n.a	n.a	Phage AS-1 had no detectable impact on the bacterial community structure.	[237]
Aeromonas salmonicida	Phage PAS-1	Isolated from water or sediment samples of the rainbow trout culture farms in South Korea.	*Myoviridae*	Phage PAS-1 showed a broad host range to other subspecies of *A. salmonicida.* (lysed 15 *A. salmonicida* subsp. *salmonicida*, 1 *A. salmonicida* subsp. *achromogenes* and 1 *A. salmonicida* subsp. *masoucida*).	n.a.	48.000	40	116.7	Phage PAS-1 efficiently reduced bacterial growth of 3 subspecies of *A. salmonicida* strain, at an MOI of 0.1, 1 and 10. *A. salmonicida* subsp. *salmonicida* growth was inhibited for 12 h after phage inoculation. Bacterial growth was not observed at an MOI of 10 000 for 48 h after phage incubation. At an MOI of 0.01, 1 and 100, the bacterial growth increased after 12, 24 and 48 h of incubation with phage, respectively.	[215, 220]
Aeromonas hydrophila	pAh1-C	Isolated from the Han River, Seoul, South Korea.	*Myoviridae*	Narrow host range (lysed only 5 of the 17 *A. hydrophila* strains tested).	-Stable at pH 7.0 -Stable at temperatures between 20 and 25 °C for 1 h.	55.000	30	60	Both phages efficiently inhibited *A. hydrophila* growth at MOIs of 0.01, 1 and 100. Phages pAh1-C and pAh6-C inhibited bacterial growth for 12 and 6 h, respectively, then phage-resistant growth increased.	[209]
	pAh6-C		*Myoviridae*	Narrow host range (lysed only 5 of the 17 *A. hydrophila* strains tested).		58.000	20	10		
Aeromonas salmonicida	Phage AS-A	Isolated from sewage network of Aveiro, Portugal.	*Myoviridae*	Narrow host range, phage only lysed the host.	n.a.	n.a.	n.a	n.a	Phage AS-A decreased significantly the bacterial growth at an MOI of 100	[51]

				No lytic activity was observed against 2 other *Aeromonas* strains (*A. caviae*, *A. hydrophila*) and 22 other strains that belong to the genus *Vibrio*, *Pseudomonas*, *Escherichia* and *Salmonella*.					(maximum inactivation of 4 log CFU/mL after 8 h). The frequency of emergence of phage-resistant mutants was 2.24×10^{-4}.	
Aeromonas hydrophila	AP1	Isolated from seawater samples of the Mattrouh seashores, Egypt.	n.a	Phage AP-1 showed a broad host range to other genera (lysed 1 *Staphylococcus aureus*, 1 *Escherichia coli* and 1 *Vibrio parahaemolyticus*).	Stable at pH 8 and at 37 °C.	>10.000	n.a	n.a	*In vitro* assays showed an increase in phage AP2 infectivity under optimized culture conditions with a 94% bacterial reduction when compared to the phage infectivity under basal conditions.	[92]
	AP2			Phage AP-2 showed a broad host range to other genera (lysed 1 *S. aureus*, 1 *E. coli*, 1 *V. parahaemolyticus*, *Vibrio alginolyticus*, *Vibrio damsela* and *Pseudomonas aeruginosa*).						
	AP3			Phage AP-3 showed a broad host range to other genera (lysed 1 *Streptococcus epidermidis* and *V. alginolyticus*).						
	AP4			Phage AP-4 showed a broad host range to other genera (lysed 1 *S. aureus*, 1 *V. damsela* and *Enterococcus faecalis*).						
Aeromonas hydrophila	Φ2	Isolated from water samples of the Saigon River in southern Vietnam.	*Myoviridae* Spounalikevirus	Both phages inhibited the growth of all the *A. hydrophila* strains tested (17 strains), but did not infect the other 27 species tested.	n.a	>20.000	10	213	Phage Φ2 adsorption assays showed that 90% of the phage particles were absorbed into the host cell after 40 min. Phages Φ2 and Φ 5 effectively inhibited *A. hydrophila* growth under laboratory conditions. The development of phage-resistant *A. hydrophila* cells increased bacterial concentration.	[95]
	Φ5						n.a	n.a		

Aeromonas salmonicida	AS-A	Isolated from sewage network of Aveiro, Portugal.	*Myoviridae*	Phages AS-A, AS-D and AS-E only infected the host (*A. salmonicida*) and could not infect the other 29 strains tested.	n.a	n.a	30	22 ± 5	Phage AS-D (maximum inactivation of 6.3 log CFU/mL) presented a higher rate of bacterial reduction than phages AS-A and AS-E (maximum inactivation of 4.0 and 6.1 log CFU/mL, respectively). The cocktail combining AS-D and AS-E was more efficient than the other cocktails and the phages individually. The use of phage cocktails, in general, decreased phage-resistant mutants.	[150]
	AS-D				Stable at 25 °C for more than 107 days. Stable at 37 °C for 21 days. Stable in pH ranging between 5.5 and 8 for more than 107 days. Phage AS-D survival was affected by sunlight exposure and UV radiation.	n.a	40	5 ± 1		
	AS-E				n.a	n.a	40	10 ± 1		
Aeromonas salmonicida	AS-szw	Isolated from water samples collected in China.	*Myoviridae*	Phage AS-szw infected only *A. salmonicida* (lysed 7 of the 7 strains tested*)* and could not infect the other 2 *A. hydrophila* tested.	n.a	229.957	40	145	Phage adsorption assays showed that 90% of the phage particles were adsorbed to *A. salmonicida* in the first 5 min. Phage AS-gz was highly effective against the host at all MOIs tested (0.01, 0.1, 1 and 10), but the MOI of 0.01 had the best results. Phage-resistant bacteria rapidly emerged shortly after treatment. The cocktail combining AS-gz and AS-yj was more efficient than the other cocktails and the phages individually.	[217]
	AS-yj			Phage AS-yi infected only *A. salmonicida* (lysed 7 of the 7 strains tested*)* and could not infect the other 2 *A. hydrophila* tested.	Phage AS-yj maintained lytic capacity at pH 5–10. Stable at temperatures between 4 and 37 °C. Decreased about 66% at 60 °C after 45 min. Phage AS-yi was completely inactivated after 15 min at 80 °C.	230.183	< 20	98		
	AS-zj			Phage AS-zj infected all *Aeromonas* species tested (lysed 7 *A. salmonicida* and 2 *A. hydrophila*).	n.a	230.023	20	86		
	AS-sw			Phage AS-sw lysed 7 *A. salmonicida* and 1 *A. hydrophila* (lysed 1 of the 2 *A. hydrophila* tested).	n.a	230.024	50	86		

	AS-gz			Phage AS-gz lysed 7 *A. salmonicida* and 1 *A. hydrophila* (lysed 1 of the 2 *A. hydrophila* tested).	Phage AS-gz maintained lytic capacity at pH 4–11.<br>Stable at temperatures between 4 and 37 °C.<br>Decreased about 90% after 15 min at 60 °C.<br>Phage AS-gz was completely inactivated after 15 min at 80 °C.	162.475	30	135		
Aeromonas hydrophila	Phage CT45P	Water samples from ponds in Can Tho and Tien Giang provinces.	n.a	n.a	90-95% phage TG25P survival at 20- 37 °C for 24 h.<br>80-85% phage TG25P survival at 50 °C for 24 h.<br>80 and 75% phage CT45P survival after 24 h at 25 and 50 °C, respectively.<br>More than 80% of the phages CT45P and TG25P survived after 24 h at pH 5-9.<br>Phage CT45P was revealed to be more pH-sensitive than phage TG25P at pH 4-5.<br>Phage TG25P remained stable in pond water for 48 h.	n.a	25	79 ±11.9	Phage TG25P effectively inactivated *A. hydrophila* (decreased approximately 3 log CFU/mL after 8 h incubation).<br>Bacterial re-growth occurred about 8 h after incubation.	[261]
	Phage TG25P						40	67±1.4		
Aeromonas salmonicida subsp. *salmonicida*	Phage ASP-1	Isolated from chopped goldfish tissues (kidney and digestive tract).	*Myoviridae*	Phage only infected the host (*A. salmonicida*) and *1 A. hydrophila.*<br>Phage infects none of the bacteria species belonging to the other nine genera tested.	Stable at pH between 4 and 11 for 1 h.<br>Phage was completely	55.000-60.000	30	16	The adsorption rate was 3.61 x10^8 PFU/mL for 3 min.	[180]

					inactivated at pH 2, 3, 12 and 14.<br>Stable for 1 h at temperatures between 4 and 50 °C.<br>Phage was completely inactivated at 65 °C.<br>Stable for 1 h at salinity 0.1-3.5%.				The bacterial growth decrease was time and MOI dependent. At an MOI of 10, the inactivation of *A. salmonicida* was faster, when compared with MOIs of 0.1 and 1.	
Aeromonas hydrophila	TG22P	Isolated from pond water samples in Can Tho and Tien Giang provinces.	n.a.	n.a.	n.a.	n.a.	25	70±10.1	*A. hydrophila* density (OD 600 nm) was gradually reduced during the first 6 h of incubation with phages TG22P, TG23P, TG25P and CT45P.<br>However, 7 h after incubation, *A. hydrophila* density increased due to the regrowth of phage-resistant bacterial strains.<br>The use of cocktails (TG25P/CT45P) did not prevent the development phage- resistant bacteria mutants.	[212]
	TG23P		n.a.	n.a.	n.a.	n.a.	25	94±15.9		
	TG25P		n.a.	n.a.	n.a.	n.a.	40	79±11.9		
	CT45P		n.a.	n.a.	n.a.	n.a.	25	67±1.4		
Aeromonas hydrophila	φZH$_1$	Isolated from sewage water.	*Podoviridae*	Both phages infected only 1 *A. salmonicida* of the 6 strains tested and	Stable at high temperatures (40 °C).<br>Phages infectivity decreased at temperatures above 40 °C.<br>Phages were		20	113	Phage adsorption assays showed that 51 and 66.8% of the phage particles of ΦZH1 and ΦZH2 were adsorbed into the host cell after 20 and 30 min, respectively.	[93]

	φZH2			did not infect any isolates of non-*Aeromonas* bacteria tested.<br><br>The phages did not infect the other 4 species tested.	completely inactivated after 5 min at 70 °C.<br><br>Phages ΦZH1 and ΦZH2 lost 50% of their infectivity after exposure to UV irradiation for 100 and 80 min, respectively.	n.a.	20	114	Both phages efficiently inhibited *A. hydrophila* growth at an MOI of 10.	
Aeromonas hydrophila	Akh-2	Isolated from water and soil samples of the Wahyeon Beach, South Korea.	*Siphoviridae*	Narrow host range, phage lysed only 4 of the 7 *A. hydrophila* strains tested and could not infect the other 23 strains tested.	Stable for 3 days at temperatures between -80 and 37 °C.<br><br>After 3 days at 45 and 50 °C, the phage titer decreased by 15 and 47%, respectively.<br><br>Phage was completely inactivated at 55 °C.<br><br>Stable for 3 days at pH between 7 and 9.<br><br>After 3 days at pH 6, 5 and 11, the phage titer decreased by 20, 75 and 65%, respectively.<br><br>Phage was completely inactivated at pH 4 and 12.	114.901	50 ± 5	139 ± 5	The results suggested that phage Akh-2 is a potential biological agent for the treatment of *Aeromonas* infections in fish.	[149]
Aeromonas hydrophila	AhyVDH1	Isolated from water samples of the Dianchi Lake (Yunnan province, China).	*Myoviridae*	Narrow host range (lysed only 1 of the 7 *A. hydrophila* strains tested).	Stable at pH between 5 and 10, but more stable at pH 6 and 7.<br><br>pH lower than 4 and higher than 11	39.175	50	274	*A. hydrophila* growth inhibition in vitro was time and MOI-dependent.<br><br>At MOIs 0.1, 1, 10, bacterial growth began to be inhibited at 2.5, 2, and 1	[214]

					resulted in the loss of phage activity.<br>Stable for 1 h at 30 °C.<br>Survival rates were 66.7 and 24.3% after 60 min of treatment at 40 and 50 °C, respectively.<br>Survival rate was less than 1% after 20 min of treatment at 60 °C.				h, respectively, and the inhibition was maintained for up to 12 h.<br>Bacterial host re-growth occurred about 12 h after incubation.	
Aeromonas hydrophila	Phage MJG	Isolated from water samples of the river in Harbin, China.	*Podoviridae*	Narrow host range. Phage can specifically lyse *A. hydrophila* (infected 4 of the 20 strains tested) but no other bacteria including other *Aeromonas* species (*A. salmonicida* subsp. *masoucida*, *A. salmonicida* subsp. *achromogenes*, *A. sobria*, and *A. caviae*).	Stable at 10, 20, 30, 40, and 50 °C for 40, 60, and 80 min.<br>Decreased 3 log PFU/mL after 80 min at 60 °C.<br>Inactivated after 40 min at 70 and 80 °C.	45.057	30	n.a	*A. hydrophila* was infected with phage at an MOI of 10, 1, 0.1, 0.001, 0.0001).	[91,394]
Aeromonas hydrophila	Phage PVN02	Isolated from striped catfish pond water samples of the Can Tho City, Vietnam.	*Myoviridae*	n.a	Stable at 15– 25 °C (retained phage activity at 70-80%).<br>Phage activity was retained at 30 °C to approximately 60% of the control.<br>Phage lost activity at 37 and 50 °C.<br>Stable at pH 7– 9.<br>pH 5, 6, 10, 11, phage titres remained at approximately 70 – 80% of the control.<br>pH 3 and 4, significantly	51.668	10	105	The phage efficiently inactivated *A. hydrophila*, however, bacterial re-growth occurred about 5 h after incubation.	[148, 213]

					reduced phage activity.					
Aeromonas hydrophila	Phage pAh6.2T	Isolated from striped catfish pond water samples of the Tien Giang Province, Vietnam.	*Myoviridae*	Narrow host range (lysed only 10 of the 17 *A. hydrophila* strains tested).<br>No lytic activity was observed against other bacterial pathogens including *A. veronii*, *Aeromonas schubertii*, *Edwardsiella ictalurid*, *Streptococcus agalactiae*, *Lactobacillus fermentum* and *Lactobacillus plantarum*.	Stable for 24 h at temperatures between 4 and 40 °C.<br>Stable for 24 h at pH between 7 and 11.<br>At pH 5, phage remained viable after 1 h and decreased to 33% after 24 h.<br>At pH 3, only 15% of the phage was still viable after 1 h and reduced to undetectable levels at 24 h.<br>Stable at salinity up to 40%.	51.780	n.a	n.a	The results suggested this phage is an effective alternative to antibiotics to control *A. hydrophila* in tilapia and possibly other freshwater fish.	[94]
Aeromonas hydrophila	Phage AHP-1	Fish tissues (digestive tract, kidney, liver, spleen, skin) of Crucian carp, *Carassius carassius.*	*Myoviridae*	Narrow host range, lysed only 2 of 6 *A. hydrophila* and 1 of 4 *A. salmonicida* strains tested.<br>No lytic activity was observed against *A. sobria*, *A. bivalvia*, *A. caviae*, *A. veronii*, *Morganella* sp., *Edwardsiella tarda*, *Enterobacter* sp., *Enterococcus faecium*, *Escherichia coli*, *Klebsiella pneumoniae*, *Streptococcus iniae*, *S. parauberis*, *Vibrio* sp. and *Lactococcus* sp.	Stable between 4 and 37 °C.<br>Decreased at 50 °C.<br>Inactivated at 65 °C.<br>Stable at pH between 7 and 10.<br>Inactivated at pH 2, 3, 12 and 14.	n.a	40	97	Phage adsorption assays showed that 81% of the phage particles were adsorbed to *A. hydrophila* in the first 25 min.<br>The bacterial inactivation was time and MOI dependent. At an MOI of 10, the phage showed higher growth inhibition against *A. hydrophila*, when compared with MOIs of 0.01, 0.1 and 1.	[181]
Aeromonas hydrophila	Phage vB-AhyM-AP1	Isolated from sewage water of the Kankanady, Mangalore, India.	*Myoviridae*	Narrow host range, lysed only 3 of 8 *A. hydrophila* isolates (37. 5%) and 1 of 12 *Aeromonas* sp. isolates.<br>No lytic activity was observed against *Vibrio mimicus*, *Vibrio vulnificus*, *V. parahaemolyticus*, *V. anguillarum* and *S. aureus*.	Stable for 60 min at temperatures between 4 and 45 °C.<br>Decreased 3.5 log PFU/mL after 60 min at 60 °C.	54.490	40	1413	Phage decreased 0.8 log units of *A. hydrophila* cells in biofilms grown on PVC coupons maintained in a low nutrient medium for 10 days.	[235]

					Stable between pH 5 and 10 for 24 h.<br>Decreased 1.3, 1.3 and 5.3 log PFU/mL after 24 h at pH 4, 11 and 12, respectively.<br>Inactivated at pH 3.<br>Stable between 0.1 and 2% salinity.<br>Decreased 0.5 log PFU/mL after 24 h at 3.5% salinity.					
Aeromonas hydrophila	Phage pAh-1	Isolated from water samples.	*Myoviridae*	Narrow host range, phage lysed only 3 of 5 *Aeromonas* isolates (*A. hydrophila*, *A. salmonocida* subsp. *masoucida* and *A. salmonicida* subsp. *salmonicida*) and no lytic activity against bacteria belonging to the other genera tested (*Bacillus cereus*, *Cronobacter sakazakii*, *E. coli*, *Staphylococcus haemolyticus*, *S. aureus* subsp. *aureus*, *V. parahaemolyticus* and *Vibrio proteolyticus*).	Stable for 1 h at 4, 20 and 40 °C.<br>Phage concentration reduced 18.9, 97.2 and 98.1% after 1 h at 60, 80 and 100 °C, respectively.<br>The optimum pH was 7.5 for 1 h.<br>Between pH 5 and 11, 90% of phage remained viable.<br>At pH 3, only 20% of phage remained viable.<br>Inactivated after 1 h at pH 1 and 12.	n.a	15	608	Phage showed bactericidal activity against *A. hydrophila* growth at an MOI of 1.	[211]
Aeromonas hydrophila	Phage Ahp2	Isolated from sewage water.	*Myoviridae*	Phage infected only 25 of the 42 *A. hydrophila* strains tested and could not infect any other species (*Acinetobacter baumannii*, *E. coli*, *Klebsiella pneumoniae*, *S. aureus*, *V. parahaemolyticus*, *Vibrio harveyi* and *Xanthomonas campestris* pv. *campestris*).	n.a	47.331	15	142	Phage adsorption assays showed that 96% of the phage particles were adsorbed to *A. hydrophila* in the first 18 min.<br>Phage Ahp2 completely lysed *A. hydrophila* AH300206 in 3.5 h at an MOI of 0.0001 and does not lysogenize its host.	[230]

Aeromonas hydrophila	Phage N21	Isolated from water samples of ponds, sewage and rivers in Nanjing.	*Myoviridae*	Phage N21 lysed 17 (22.67%) of 75 *A. hydrophila* isolates, 1 of 12 *A. caviae* strains tested and 1 of 85 *A. veronii* strains tested. Did not infect the other species: *A. bestiarum*, *A. sobria*, *A. media*, *A. salmonicida*, *A. jandaei* and *A. aquariorum.*	Stable for 1 day at 4 and 30 °C.<br>Inactivated after 100 and 60 min at 50 and 60 °C, respectively.<br>Stable for 2 h at pH between 5 and 11.<br>After 2 h at pH 4 and 12, about 75 and 20% of the phage, respectively, remained viable.<br>Inactivated after 2 h at pH 3.	n.a	15	316	All phages efficiently reduced *A. hydrophilia* growth at an MOI of 0.01, 0.1, 1 and 10.<br>Bacterial re-growth occurred about 12 h after incubation with phages N21, W3 and Y71.<br>Phages G65 and Y81 showed a considerable bacterial killing effect and potential in preventing the formation of *A. hydrophila* biofilm; phages G65, W3 and N21 were able to scavenge mature biofilms effectively.	[236]
	Phage W3		*Myoviridae*	Phage W3 lysed 16 (21.33%) of 75 *A. hydrophila* isolates, 1 of 12 *A. caviae* strains tested, 3 of 85 *A. veronii* strains tested and 1 *A. bestiarum.* Did not infect the other species: *A. sobria*, *A. media*, *A. salmonicida*, *A. jandaei* and *A. aquariorum.*	Stable for 1 day at 4 and 30 °C.<br>Stable for 100 min at 40 °C.<br>Inactivated after 20 min at 60 °C.<br>Stable for 2 h at pH between 4 and 10.<br>Stable for 100 min at 40 °C.<br>After 2 h at pH 3, only 15 % of the phage was still viable.<br>Inactivated after 2 h at pH 12.	n.a	15	160		
	Phage G65		*Myoviridae*	Phage G65 lysed 16 (21.33%) of 75 A. *hydrophila* isolates, 1 of 12 *A. caviae* strains tested and 3 of 85 *A. veronii* strains tested). Did not infect the other species: *A. bestiarum*, *A. sobria*, *A. media*, *A. salmonicida*, *A. jandaei* and *A. aquariorum.*	Stable for 1 day at 4 and 30 °C.<br>Stable for 100 min at 40 °C.<br>Inactivated after 20 min at 60 °C.	n.a	15	210		

					After 2 h at pH between 4 and 11, approximately 75 % of the phage was still viable.<br>After 2 h at pH 3 and 12, only 25 and 40% of the phage, respectively, was still viable.					
	Phage Y71		*Podoviridae*	Phage Y71 lysed 15 (20%) of 75 *A. hydrophila* isolates and 1 of 85 *A. veronii* strains tested. Did not infect the other species: *A. caviae*, A. *bestiarum*, *A. sobria*, *A. media*, *A. salmonicida*, *A. jandaei* and *A. aquariorum*.	Stable for 1 day at 4 and 30 °C.<br>Stable for 100 min at 40 °C.<br>Inactivated after 50 min at 60 °C.<br>Stable for 2 h at pH between 5 and 10.<br>After 2 h at pH 3 and 11, only 20 and 10% of phage, respectively, was still viable.<br>Inactivated after 2 h at pH 12.	n.a	15	200		
	Phage Y81		*Podoviridae*	Phage Y81 lysed 17 (22.67%) of 75 *A. hydrophila* isolates and 1 the 85 *A. veronii* strains tested. Did not infect the other species: *A. caviae*, *A. bestiarum*, *A. sobria*, *A. media*, *A. salmonicida*, *A. jandaei* and *A. aquariorum*.	Stable for 1 day at 4 and 30 °C.<br>Stable for 100 min at 40 °C.<br>Inactivated after 20 min at 60 °C.<br>Stable for 2 h at pH between 4 and 10.<br>Only 25 and 15% of the phage remained viable at pH 3 and 11, respectively.	n.a	15	220		

					Inactivated after 2 h at pH 12.					
Aeromonas hydrophila	Phage AhFM4	Isolated from water samples of the rivers of Karnataka, India	*Myoviridae*	Narrow host range. Phage infected only *A. hydrophila* (lysed 14 of the 60 strains tested).	Decreased 3 log PFU/mL after 60 days at 0 and 4 °C. Decreased 5 log PFU/mL after 45 days at 28 °C. Inactivated after 60 days at 37 °C. Stable for 60 days at pH between 5 and 8. Decreased after 60 days at pH 2, 4, 10 and 12. Stable for 60 days with salinity between 0.5 and 2%. Decreased after 60 days at 0.1 and 3.5 % salinity.	n.a	15	152 ± 27	Phage AhFM4 showed a high adsorption efficacy with a 96% adsorption within 30 min to the host cells and no free phages were detected in the supernatant after 40 min. Phage AhFM5 showed low adsorption efficacy where only 70% of phage was adsorbed within 30 min to the host cells and no free phages were detected in the supernatant after 55 min.	[231]
	Phage AhFM5			Narrow host range. Phage infected only *A. hydrophila* (lysed 12 of the 60 strains tested.			25	112 ± 13		

Table S3. In vivo studies of phage therapy for controlling *Aeromonas* sp. in fish.

Pathogen	Species	Phage	Phage source	Phage administration	Outcome	Reference
Aeromonas hydrophila	Loach (*Misgurnus anguillicaudatus*)	Phage AH1	Isolated from pond and sewage water.	*A. hydrophila* was infected with phage AH1 at different MOIs (0.001, 0.01, 0.1, 1 and 2) in sterilized water.	3 h after phage administration at an MOI of 2, *A. hydrophila* had completely lost its infectivity and mortality in the treated fish.	[174]

				The infected bacteria were centrifuged, suspended in Ringer's solution and injected to loach *M. anguillicaudatus.*	*A. hydrophila* was also effectively eliminated at MOIs of 0.01, 0.1 and 1. At an MOI of 0.001, the bacterial infectivity and mortality were reduced by 40%.	
Aeromonas salmonicida	Brook trout (*Salvelinus fontinalis*)	Phage HER 110 (*Myoviridae*)	Isolated from water samples of the La Petite Mouge River in France.	*A. salmonicida* (10^8 CFU/mL) was added to aquariums (day 1). On days 5 and 6, phage HER 110 (10^9–10^{10} PFU/mL) was added to the aquariums.	In the aquariums with brook trout, the *A. salmonicida* concentration (10^8 CFU/mL) decreased 6 log CFU/mL in 3 days at an MOI of 1.<br>The phage addition delayed the onset of furunculosis in brook trout by 7 days.<br>The fish mortality rates have been reduced from 100% to 10% after 45 days.<br>Tests revealed that more than one phage infected the bacterial strain and phage HER 110-resistant mutants were sensitive to other phages. Phage-resistant bacteria had a slower generation time than the original strain and very low replanting success in TSA.	[218]
Aeromonas salmonicida subsp. *salmonicida*	Juvenile Atlantic salmon (*Salmo salar*)	Phages O, R and B	Phages B and O were isolated from a fish farm effluent (UK and France, respectively).<br>Phage R was isolated from a reservoir (France).	Fishes were administered with bacteria and the mixture of phages O, R and B (1.9×10^8 PFU/fish) by intraperitoneal injection immediately and 24 h after bacterial infection.<br>To evaluate the effect of phage treatments (oral, bath and intraperitoneal injection) with chemotherapy using an indirect cohabitation challenge, fish were inoculated by intraperitoneal injection with *A. salmonicida* subsp. *salmonicida* (8.28×10^4 CFU fish^{-1}) and treated with the mixture of phages O, R and B (10^5-10^9 PFU/fish) or antibiotic (10 mg^{-1} kg^{-1} oxolinic acid).	Fish injected with the phage combination, immediately after bacterial inoculation, died at a significantly slower rate than those without phage treatment or treated 24 h after inoculation.<br>However, 100% mortality was observed at the end of the assays.<br>When the effects of phage treatments were compared (oral, bath and intraperitoneal injection) with chemotherapy using an indirect cohabitation challenge, no protection was offered by the phage treatments, but significant protection was offered by the oxalinic acid treatment.<br>Phage-resistant bacteria were isolated from dead juveniles in all treatment groups.	[219]

Aeromonas hydrophila	Loaches (*Misgurnus anguillicaudatus*)	Phages pAh1-C and pAh6-C (*Myoviridae*)	Isolated from the water of the Han River	Fishes were infected with two different bacterial concentrations (10^6 CFU/mL and 10^7 CFU/mL) and each phage (10^7 PFU/mL) was administered through intraperitoneal injection or incorporated in the feed for oral administration.	The administration of both phages to cyprinid loaches resulted in noticeable protective effects, with an increased survival rate against *A. hydrophil*a infection.	[209]
Aeromonas hydrophila	Tilapia (*Oreochromis niloticus*)	Phage UP87	Isolated from sewage water samples (Barangay Ugong, Pasig City)	Fish were inoculated by intraperitoneal injection with *A. hydrophila* at a LD_{100} and phage UP87 at 10^7 PFU/mL.	There were no significant differences between the group treated with oxytetracycline and the group treated with phage UP87. Phage application let to a reduction of 100% in mortality during the 72 h of trial.	[277]
Aeromonas salmonicida subsp. *salmonicida*	Juvenile rainbow Trout (*Oncorhynchus mykiss*)	Phage PAS-1 (*Myoviridae*)	Isolated from water or sediment samples of the rainbow trout culture farms in Korea.	*A. salmonicida* subsp. *salmonicida* was inoculated intramuscularly in fish (2.5 x 10^2 CFU/fish) and immediately injected with the phage (2.4 x 10^6 PFU/fish).	A 26% survival rate was verified in phage administered fish. The fish that remained alive after 14 days did not reveal any type of ulceration.<br>The control group (only bacteria) presented 100% mortality 2 days into the trial.	[220]
Aeromonas salmonicida	Senegalese sole (*Solea senegalensis*)	Phage AS-(*Myoviridae*)	Isolated from sewage network of Aveiro, Portugal.	Phage (10^{10} PFU/mL) was added to seawater tanks with artificially contaminated (10^8 CFU/mL of *A. salmonicida*) juveniles of *S. senegalensis*.	The phage only infected *A. salmonicida* and did not infect the other 24 strains tested.<br>After 6 h of treatment, the phage inhibited *A. salmonicida* growth in both batch cultures and seawater in the presence of fish (≈4 and 2.5 log PFU/mL, respectively).<br>No mortality was detected 72 h after the fish exposed to *A. salmonicida* were treated with the phage. The untreated fish presented a 36% mortality.<br>No significant phage impact was detected on the bacterial communities of the aquaculture water. The bacterial community associated with the fish intestinal tract was moderately affected by phage addition.	[51]
Aeromonas hydrophila	Nile tilapia (*Oreochromis niloticus*)	Phage AP2	Isolated from seawater samples of	Phage AP2 (MOI 1 and 0.5) was added to seawater tanks with juveniles of *O. niloticus* artificially contaminated with *A. hydrophila* (28 x 10^4 CFU/mL).	An *A. hydrophila* reduction in the rearing water was observed after 24 h.	[92]

			the Mattrouh seashores, Egypt.		The morphological and histological examination of the tested liver and gills tissues of the Nile tilapia proved the promising effect of phage AP2 to treat motile *Aeromonas* septicemia induced by *A. hydrophila.*	
Aeromonas hydrophila	Striped catfish (*Pangasianodon hypophthalmus*)	Phages Φ2 and Φ 5 (*Myoviridae*)	Isolated from water samples of the Saigon River in southern Vietnam.	Fishes were infected intraperitoneally with *A. hydrophila* (10^6 CFU/fish) and immediately injected with the phage cocktail (MOI 0.01, 1 and 100).	The fish contaminated only with bacteria had higher mortality rates (82%) than the fish without bacteria nor phages.<br>No mortality was observed in the groups at an MOI of 100. At MOIs 1 and 0.01, the mortalities were 45 and 68%, respectively.	[95]
Aeromonas hydrophila	Nile tilapia (*Oreochromis niloticus*)	Phages φZH_1 and φZH_2 (*Podoviridae*)	Isolated from sewage water.	Phages (MOI 2.1) was added to seawater tanks with *O. niloticus* artificially contaminated with *A. hydrophila.*	Phages reduced the percentage of mortality from 68 to 18% after 15 days of treatment.<br>The bacterial concentration in the water reduced from 4.18×10^{13} to 7.5×10^{7} CFU/mL after three days of treatments.	[93]
Aeromonas hydrophila	Zebrafish (*Danio rerio*)	Phage pAh-1 (*Myoviridae*)	Isolated from water samples.	Fishes were simultaneously injected intraperitoneally with *A. hydrophila* (10^6 CFU/fish) and phage (10^7 PFU/ fish, MOI of 10).	*A. hydrophila*-injected fish (without pAh-1 treatment) showed dark internal areas due to abdominal haemorrhage and pale skin with less pigmentation compared to that of pAh-1-treated.<br>Fish challenged with *A. hydrophila* had cumulative mortality 48 h after post-infection.<br>Fish treated with pAh-1 showed a cumulative mortality rate lower (56.67±35.11%) than the fish challenged with *A. hydrophila* and not treated with phage (96.67± 5.77%).	[211]
Aeromonas hydrophila	Loach (*Misgurnus anguillicaudatus*)	Phage Akh-2 (Siphoviridae)	Isolated from water and soil samples of the Wahyeon Beach.	Phage (10^8 PFU/mL) was added to seawater tanks with *M. anguillicaudatus* artificially contaminated with *A. hydrophila* (10^7 CFU/mL).	Phage reduced the percentage of mortality from 100 to 57% after 96 h of treatment.	[149]

Aeromonas hydrophila	Nile tilapia (*Oreochromis niloticus*)	Phage PAh4 (*Myoviridae*)	Isolated from water samples of Ponds. (Nong Khon Farm (Ubon Ratchathani, Thailand).	Phage was applied in food supplement at 10^5, 10^6, 10^7 and 10^8 PFU/g of diet. Fish were infected with *A. hydrophila* UR1 by intraperitoneal injection at LD_{50} dose. Fish were then fed with different diets for 10 days.	The increase in phage dose (10^{-5} to 10^8 PFU/g of diet) led to a reduction in cumulative mortality from approximately 20% to nearly 0%.<br>There was no difference in the mortality rates of fish fed with oxytetracycline and phages at 10^8 PFU/g of diet.	[276]
Aeromonas hydrophila	Rainbow trout (*Oncorhynchus mykiss*)	Phage MJG (*Podoviridae*)	Isolated from water samples of the river in Harbin, China.	Fish was injected intraperitoneally with *A. hydrophila* (10^8 CFU/fish) and treated with a single dose of MJG (3.2×10^6 PFU/fish) administered intraperitoneally at 2 h post bacterial infection or immersed in water for 15 min with phage at a concentration of 3.2×10^6 or 3.2×10^5 PFU/ml.	Phage MJG administered through injection or immersion completely protected the fish from death due to *A. hydrophila* infection.<br>The expression levels of pro-inflammatory cytokines (IL-8 and IL-1β) in the spleen of MJG-treated fish were significantly higher than those in PBS-treated fish at 1- or 2-days post-infection but significantly lower than those in PBS treated fish at 3 days post-infection.<br>MJG treatment restored liver tissue damages and abolish the clinical signs of infection.	[91, 394]
Aeromonas hydrophila	Striped catfish (*Pangasianodon hypophthalmus*)	Phage PVN02 (*Myoviridae*)	Isolated from striped catfish pond water samples of the Can Tho City, Vietnam.	In the first day, fish were fed phage-sprayed pellets (10^4, 10^5 and 10^6 PFU/g). On the second day, the fish in the tanks received the bacterial (10^3, 10^4, 10^5, 10^6 and 10^7 CFU/ml) challenge by immersion for 24 h.	Phage PVN02 protected fish from the disease by significantly reducing the mortality rate when compared to the control without phages.<br>The mortality rate of bacteria-infected fish (10^7 CFU/mL) was significantly reduced to 8.33 ± 2.9% or 16.67 ± 2.9% (absence phage mortality rate was 68.3 ± 2.9%) at the phage dose of log 6.2 ± 0.09 or log 4.2 ± 0.09 PFU/g.	[148, 213]

Aeromonas hydrophila	Nile tilapia (*Oreochromis niloticus*)	Phage pAh6.2T (*Myoviridae*)	Isolated from striped catfish pond water samples of the Tien Giang Province, Vietnam.	Phage (10^6 and 10^7 PFU/mL) and *A. hydrophila* (10^7 CFU/mL) were added to tanks with fish.	Phage treatments significantly improved fish survival with relative percent survival of 73.3 and 50% for an MOI of 1.0 and 0.1, respectively.<br>Significant reduction of bacterial counts in rearing water at 3 h (6.7 ± 0.5 to 18.1 ± 6.98 folds) and in fish liver at 48 h post-treatment (2.7 ± 0.24 to 34.08 ± 26.4 folds) was observed in phage treated groups.<br>Surviving fish developed a specific antibody (IgM) against the challenged *A. hydrophila*.	[94]
